# Techno-economic optimization and sensitivity analysis of a hybrid renewable microgrid for local market electrification in developing countries

**DOI:** 10.1038/s41598-026-58196-5

**Published:** 2026-06-16

**Authors:** Diganto Biswas, Md. Feroz Ali, Md. Shafiul Alam, Obaidullah Obaidi, Mohammad Ali, Imil Hamda Imran

**Affiliations:** 1 Department of Electrical and Electronic Engineering, Pabna University of Science and Technology, Pabna, 6600 Bangladesh; 2 Department of Electrical Engineering, College of Engineering, King Faisal University, Al Ahsa, 31982 Saudi Arabia; 3 Department of Energy Engineering, Faculty of Engineering, Kabul University, Kabul, 1006 Afghanistan

**Keywords:** Hybrid renewable microgrid, Market electrification, Techno-economic optimization, Sensitivity analysis, Rural Bangladesh, Energy and society, Energy science and technology, Engineering

## Abstract

Reliable electricity supply is essential for sustaining commercial activity in rural markets, yet energy planning studies in Bangladesh predominantly rely on residential load assumptions that inadequately represent market-specific demand patterns. This study presents a comprehensive techno-economic and environmental assessment of a grid-interactive hybrid renewable microgrid for local market electrification, using Nazipur Noor market as a case study. A realistic market-based load profile capturing pronounced daytime demand concentration, peak coincidence, and seasonal variability is developed. The proposed system integrates solar photovoltaic (PV), wind turbine (WT), biogas generation (BioGen), battery energy storage (BESS), and utility grid support, optimized using HOMER Pro with hourly resource and demand data. Among 1,056 simulated configurations, the optimal PV–WT–BioGen–BESS–Grid system achieves a net present cost (NPC) of USD 91,255.5 and cost of energy (COE) of USD 0.0167/kWh, with 90.97% renewable fraction while limiting grid purchases to 8.51%. Solar PV, wind, and biogas contribute 48.4%, 25.9%, and 17.2% of annual generation, respectively. The system reduces CO₂ emissions by 83.5% compared with grid-dependent scenarios. Sensitivity analyses identify wind speed, hub height, and electricity sellback rate as dominant performance factors. Results demonstrate renewable-rich microgrids offer cost-effective, scalable solutions for sustainable market electrification in Bangladesh and similar developing regions.

## Introduction

The demand for electricity globally is rising at a rapid rate, especially in line with population growth, urbanization, and economic development. Population growth, rapid urbanization, and economic development have led to a steady growth in electricity demand in Bangladesh, which is putting tremendous pressure on the national power sector of Bangladesh^1,2^. While the country is nearing universal electrification, power security and price volatility remain problematic due to the ongoing predominance of fossil fuel–based generation^3^. While the overall installed generation capacity has reached more than 30 GW, renewable energy contributes only a small share^4^. In 2025, Bangladesh’s cumulative renewable capacity reached approximately 1,562 MW, primarily from solar PV, with minor contributions from wind, hydropower, and biogas^5^. Expanding renewable-based generation is therefore critical for enhancing energy security, reducing emissions, and supporting sustainable development. Decentralized and community-based energy assessments are essential for capturing demand characteristics and enabling effective energy planning in semi-urban and rural regions of Bangladesh^6^.

Access to reliable and cheap electricity is one of the major factors in improving economic productivity and development in a region or improving the quality of life in a region or country^7,8^, especially in an emerging country or region. Bangladesh is experiencing a rapid growth in the demand for electricity in recent times due to population growth, increased commercial activities, and rising per capita energy consumption over the last decade or so^6,9^. Despite the fact that Bangladesh has achieved national electrification, the power sector of Bangladesh has been made to suffer from voltage instability, interruptions in electricity supply, and seasonal load stress, which results in poor industrial output and economic development in the region or country^10,4,1^. These challenges are more pronounced outside major urban centers, where grid infrastructure is often weaker and demand patterns are increasingly shaped by productive and commercial electricity use rather than household consumption alone.

However, despite the national energy planning frameworks that focus on the expansion and diversification of the generating capacity, the reliance on fossil fuels makes the electricity sector susceptible to fuel price and import risks^11,12^. Renewable energy deployment has increased in recent years; however, many off-grid and donor-supported renewable projects have struggled to deliver sustained performance due to shortcomings in system sizing, maintenance structures, and long-term financial viability^5,13^. At the same time, rapid urbanization, rising energy demand, and climate-related vulnerabilities further complicate the transition toward environmentally sustainable energy systems in Bangladesh^14,15^. While national decarbonization strategies highlight the importance of expanding renewable energy generation, the current renewable share remains modest relative to the country’s technical potential, indicating a significant gap between policy ambition and implementation outcomes^16,17^.

Electricity demand growth in Bangladesh is increasingly driven by non-residential consumption, particularly in rural and semi-urban areas where local markets function as economic and social hubs^18^. These markets support agricultural trade, small enterprises, cold storage, milling operations, and service-based activities that depend on reliable electricity supply. Unlike residential demand, market electricity consumption is characterized by strong daytime concentration, high peak coincidence among commercial appliances, and seasonal variability linked to agricultural cycles and local economic activity^19,20^. However, most existing techno-economic studies of HRES in Bangladesh focus primarily on residential or aggregated rural loads, often relying on simplified demand representations that do not adequately capture the operational complexity of market-based electricity consumption^21,22^. As a result, system designs derived from such analyses may underestimate peak demand, misrepresent storage requirements, and overlook critical grid-interaction behavior.

HRES, integrating solar PV, wind turbines, biogas or biomass-based generation, BESS, and grid support, offer a promising pathway for addressing these challenges by balancing renewable intermittency and improving supply reliability^23,24^. The addition of energy storage is a significant step in addressing the fluctuations of renewable energy sources and the need for the national grid during peak hours^25^. Bangladesh possesses favorable solar and wind resources across much of its territory, while biogas-based generation provides a dispatchable renewable option well suited to rural contexts^2,26^. Nevertheless, the practical feasibility and economic performance of hybrid microgrids depend strongly on accurate load characterization, realistic modeling of power electronic converters, and systematic evaluation of system behavior under resource, economic, and grid-reliability uncertainties—issues that remain insufficiently explored in the existing literature^27,28^.

Recent studies have extensively investigated HRES as a pathway to enhance energy security, reduce fossil-fuel dependence, and improve electricity reliability in Bangladesh, with particular emphasis on rural and residential electrification. Ali et al. (2025)^29^ optimized a grid-connected residential PV–wind–BESS microgrid using HOMER Pro and reported a COE of $0.035/kWh with a low NPC of $18,266, achieving 49.6% CO₂ emission reduction and confirmed voltage–frequency stability through MATLAB simulations. In a broader national context, Raihan et al. (2025)^30^ reviewed Bangladesh’s renewable energy resources and reported a current renewable capacity of 950 MW, highlighting solar, wind, and bioenergy as key contributors for off-grid electrification, while emphasizing policy and financing challenges.

As summarized in Table 1, previous studies have demonstrated the potential of hybrid renewable energy systems in improving cost efficiency and reducing emissions. However, most studies focus on residential or generalized rural loads and often neglect the distinct characteristics of commercial demand in local markets. In addition, limited attention has been given to realistic load modeling, grid-interactive behavior, and comprehensive sensitivity analysis. These limitations highlight the need for a more application-specific and data-driven approach, which is addressed in this study.

**Table 1:** Comparative analysis of recent hybrid microgrid studies.

Ref.	Technology Used	Optimization Tool	Key Results	Limitations
Buragohain et al. (2021)^31^	PV–Biogas	Experimental	LCOE = $0.21/kWh; CO₂ reduction = 104.59 tCO₂	Small-scale system; lacks grid interaction
Sarkar et al. (2019)^32^	PV–Wind–Biogas–VRFB	HOMER + PSCAD	Zero LPSP achieved	No economic comparison across multiple configurations
Ali et al. (2025)^2^	PV–WT–BioGen–BESS–Grid	HOMER Pro	COE = $0.0256/kWh; RF = 89.04%	Uses generalized rural load, not market-specific
Mojumder et al. (2024)^4^	Hybrid + Grid	HOMER Pro	COE = $0.0436/kWh	Limited sensitivity analysis
Das et al. (2021)^6^	PV–Diesel–Pump-Hydro	Simulation-based	COE = $0.24–0.27/kWh	Fossil fuel dependency remains
Alshammari et al. (2018)^33^	PV–Biomass	HOMER	COE = $0.099/kWh	Standalone system; no grid support
Alshammari et al. (2020)^34^	Wind–Biomass–PV–Battery	PSO + Harmony Search	COE = $0.254/kWh	High cost; no grid interaction
Molu et al. (2023)^35^	Hybrid Standalone System	HOMER	COE = $0.1981/kWh; IRR = 9.09%	Residential focus; lacks commercial demand modeling

On an overall note, grid interactive systems are economically more viable. In their study on evaluating a grid-tied PV-biogas-battery hybrid system in Gaza, Al-Najjar et al. (2022)^36^ reported a renewable fraction of 64.3% with a high COE of 0.438 $/kWh. This indicates how difficult it is to operate under extremely limited grid conditions. In another study on evaluating a PV-wind-biomass-battery system in Semnan, Iran, Sadeghi et al. (2024)^37^ reported a COE of 0.201 $/kWh with a reduction in CO2 emissions by 97%. The economic viability of biomass-based systems has been well established by Irshad et al. (2024)^38^, who showed that through advanced technology in biomass conversion using pyrolysis technology, the COE could be reduced to 0.027 $/kWh with a renewable fraction of 92%.

Furthermore, hybrid microgrids have also been applied in urban and institutional areas. Though high investment costs remained an issue, Abdelsattar et al. (2024)^39^ have modeled a grid-connected hybrid energy system in Hurghada, Egypt, and have achieved 85% renewable energy penetration with an LCOE of $0.07/kWh. By using HOMER software, Aykut et al. (2020)^40^ have studied a university-level grid-connected hybrid energy system, and an NPC of $5.62 million has been achieved with a COE of $0.067/kWh for an ideal configuration consisting of 1500 kW wind and 1000 kW biomass. In a similar context, Serat et al. (2024)^41^ have studied a grid-connected hybrid energy system consisting of PV, wind, genset, and grid, and an LCOE of $0.0172/kWh, one of the lowest in comparison with existing results, has been achieved with 94.8% renewable energy penetration. Though some operational issues have been pointed out due to the availability of biogas, Kasaeian et al.^42^have studied a grid-connected hybrid energy system consisting of PV, diesel, and biogas, and have verified its efficacy in reducing diesel consumption and emissions.

To address the identified research gaps, this study is guided by the following research questions: (i) How can a realistic market-specific load profile improve the accuracy of hybrid microgrid design compared to conventional residential assumptions? (ii) What is the optimal configuration of a PV–WT–BioGen–BESS–Grid system for market electrification in terms of cost, reliability, and sustainability? (iii) Which techno-economic parameters most significantly influence system performance under varying resource and economic conditions?

The novelty of this study lies in the development of a high-resolution market-oriented load model that captures the unique characteristics of rural commercial demand, including peak coincidence, daytime concentration, and seasonal variability. Unlike existing studies that rely on residential or aggregated load assumptions, this work integrates realistic commercial demand modeling with a multi-source hybrid system and grid interaction. In addition, comprehensive sensitivity and uncertainty analyses are performed to identify the dominant parameters affecting system performance.

The main contributions of this study are summarized as follows: (1) Development of a detailed and realistic commercial load profile for a rural market in Bangladesh; (2) Techno-economic optimization of a grid-connected PV–WT–BioGen–BESS hybrid microgrid using HOMER Pro; (3) Evaluation of 1,056 system configurations to determine the most cost-effective and sustainable solution; (4) Comprehensive sensitivity analysis identifying key influencing factors such as wind speed, hub height, and electricity sellback rate; (5) Demonstration of a scalable and practical framework for market-level electrification in developing regions.

## Materials and methods

The study includes the following steps: the selection of the site, the load assessment, the evaluation of the renewable resource, the microgrid design, the techno-economic optimization, the environmental evaluation, and the performance evaluation. The overall methodology framework adopted in this study is presented in a systematic manner to clearly describe the sequential research procedure, including site selection, market load assessment, renewable resource evaluation, hybrid microgrid configuration, techno-economic optimization, sensitivity analysis, and performance evaluation. Furthermore, the methodological workflow diagram is incorporated to provide a clearer representation of the integrated simulation and optimization process used in this research.

### Site location

**Fig. 1:**
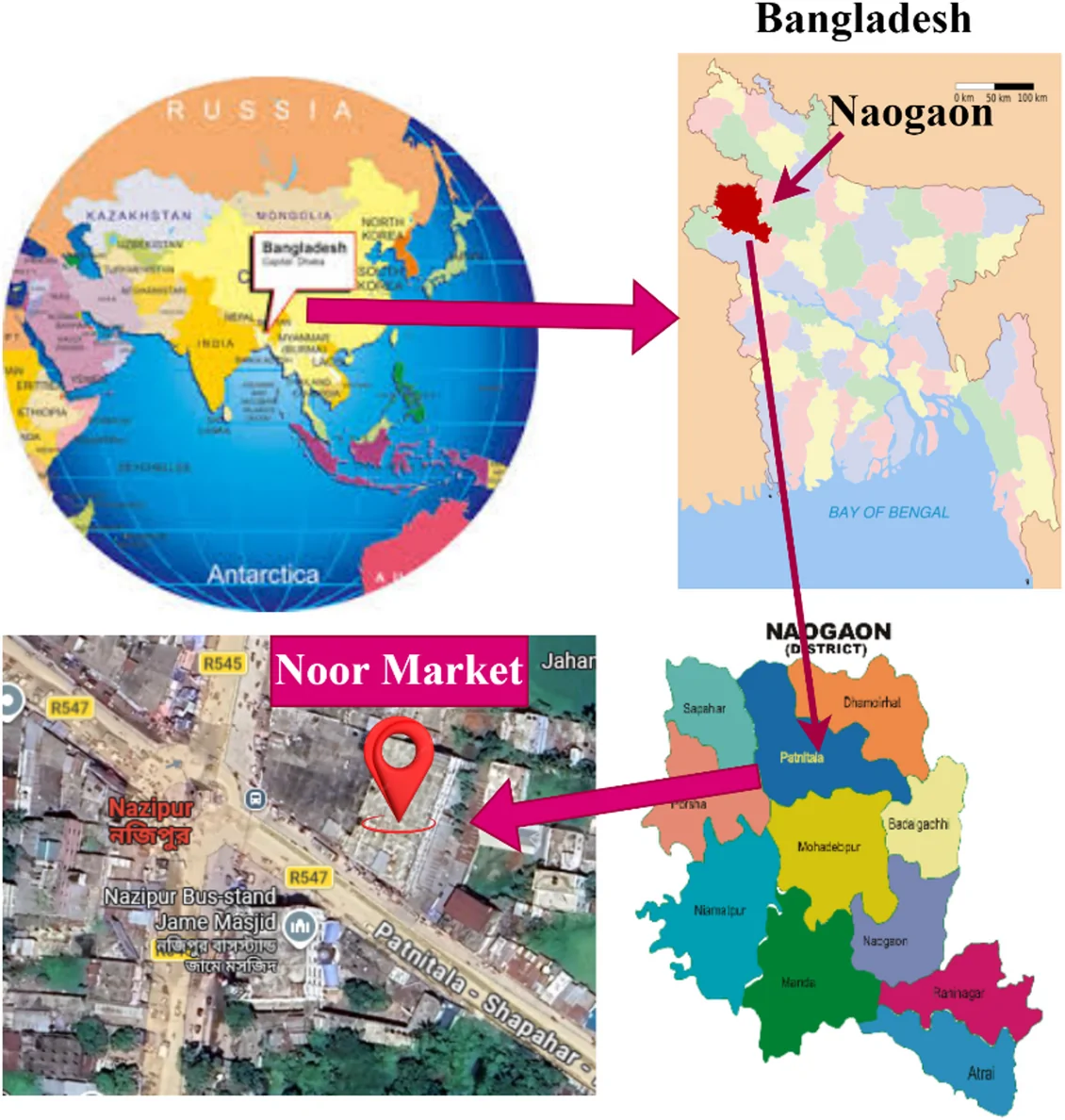
Location map illustrating the geographical position of the study area (created by using Draw.io software, version v26.1.1).

Figure 1 depicts the hierarchical geographic position of the study area created by Draw.io software^43^, identifying Nazipur Noor market within Nazipur Union, Patnitala Upazila, Naogaon District, Rajshahi Division, Bangladesh. The figure provides spatial context from the national to local scale and marks the exact coordinates of the selected market. It also depicts the immediate market surroundings, illustrating its role as a localized commercial hub. This spatial representation supports the selection of the site as a representative rural market for evaluating grid-interactive hybrid microgrid electrification.

### Load profile modeling

**Fig. 2:**
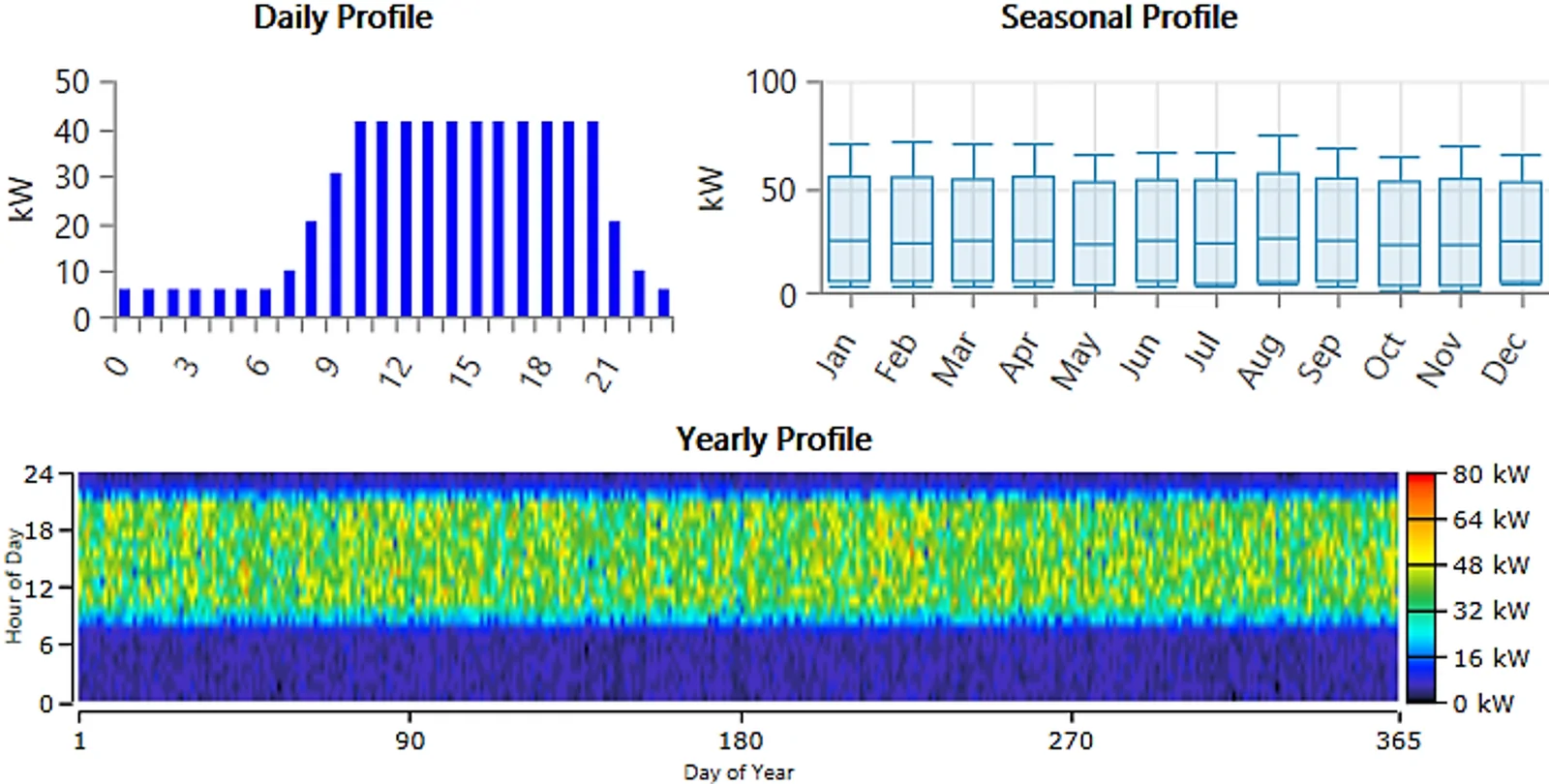
Daily, seasonal, and annual electricity demand patterns of Nazipur Noor Market.

Figure 2 shows the daily, seasonal, and annual electricity demand profiles of the Nazipur Noor market. The load profile shows a pronounced daytime demand concentration, reflecting market operating hours, with a peak load of 74.5 kW, an average demand of 24.84 kW, and an average daily energy consumption of 596.06 kWh. Seasonal variations are moderate, indicating relatively consistent commercial activity throughout the year. This detailed load characterization captures realistic market behavior and provides a reliable basis for hybrid microgrid sizing and optimization.

The market load profile was developed using a bottom-up approach based on appliance-level assessment of different commercial entities within the market, including shops, refrigeration units, lighting, milling equipment, and service facilities. Each appliance category was characterized by its rated power, operating duration, and time-of-use pattern. Hourly demand profiles were then generated by aggregating the contributions of all appliances, ensuring accurate representation of peak coincidence and daytime load concentration. This approach captures realistic demand variability and avoids the simplifications associated with static or averaged load models commonly used in previous studies. Unlike conventional static load models, which typically assume constant or averaged demand over time, the proposed market-based load profile incorporates temporal variability, appliance simultaneity, and operational diversity across different commercial activities. This enables more accurate estimation of peak demand, storage requirements, and system sizing.

### Meteorological analysis

For HOMER simulation, data on sunlight, clarity of the sky, temperature, and wind speed was retrieved from the NASA Surface Meteorology and Solar Energy Database^44^. The simulation uses different green energy options, and data is retrieved from all sorts of places. Homer Pro uses NASA’s long-term climate averages, usually spanning 22 years from 1983 to 2005, as the default data source for temperature, wind speed, and solar radiation data, and averages it out for the specified location^45,46^.

#### Analysis of solar irradiation and atmospheric clearness index

**Fig. 3:**
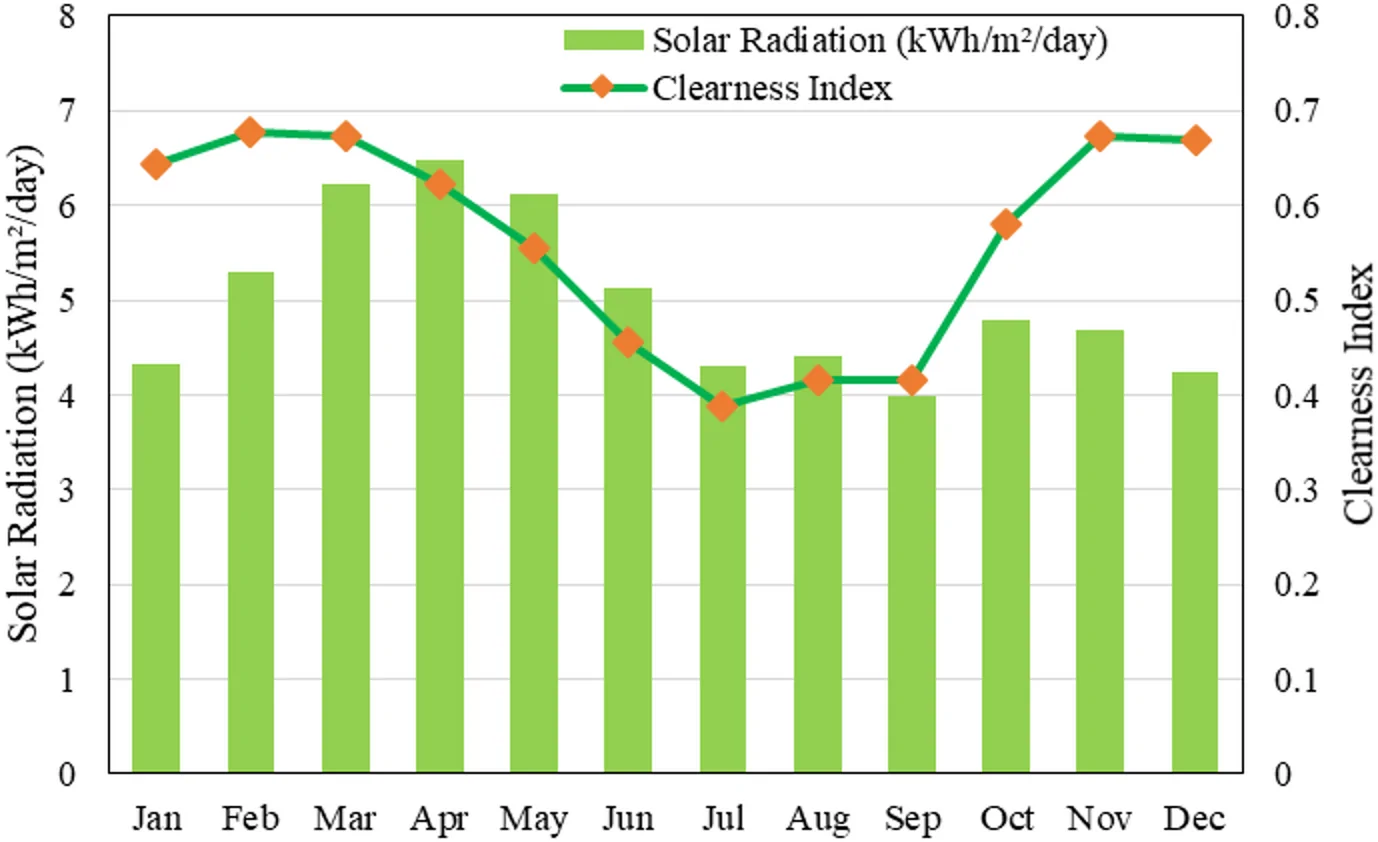
Solar radiation levels and corresponding clearness index observed at the study site.

Figure 3 graphically shows the monthly variability of the clearness index and the average daily solar radiation. The clearness index is 0.677 in February. July has the minimum clearness index of approximately 0.388, indicating reduced atmospheric transparency during the monsoon period. This reduction is primarily due to increased cloud cover during the monsoon season. This indicates that the transparency of the atmosphere changes seasonally and also changes throughout the year. The solar radiation was also seen to have its maximum in April at 6.47 kWh/m²/day. March had the second-highest at 6.22 kWh/m²/day, and May had the third-highest at 6.12 kWh/m²/day. The lowest solar radiation was seen in September at 3.99 kWh/m²/day. However, the relative low levels of solar radiation were also seen in the monsoon season. For July, the solar radiation was seen to be at 4.31 kWh/m²/day, and for August, it was seen to be at 4.41 kWh/m²/day due to the presence of clouds. The solar energy system design will thus be able to take care of the seasonal changes and will also be able to draw power from either wind energy or biogas to be able to supply power throughout the year^47,48^.

#### Temperature

**Fig. 4:**
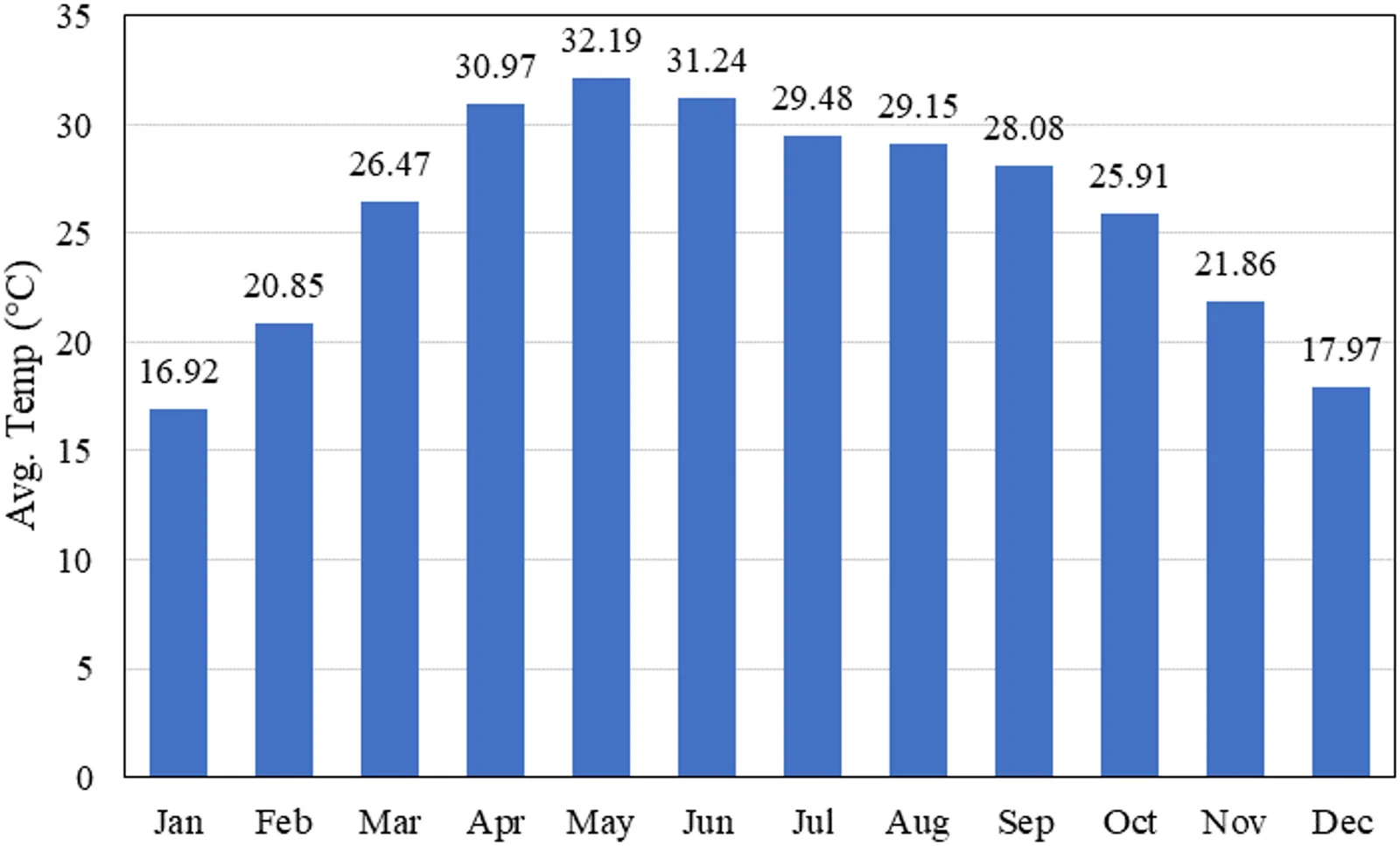
Monthly changes in ambient temperature at the selected location.

Figure 4 illustrates the monthly average temperature profile for the study site. The temperature gradually increases from 16.92 °C in January to 20.85 °C in February and finally to 26.47 °C in March. This shows the transition from winter to the warmer spring season. The temperature gradually increases and peaks at 30.97 °C in April. The temperature increases further and peaks at 32.19 °C in May. This is the hottest season of the year. There is a slight decrease in temperature from June (31.24 °C), followed by 29.48 °C in July and 29.15 °C in August. This shows the effect of the monsoon season. As the year progresses towards autumn season, the temperature gradually decreases to 28.08 °C in September and further to 25.91 °C in October. During the late autumn and winter seasons, the temperature decreases to 21.86 °C in November and finally to 17.97 °C in December. The temperature pattern shows the typical temperature pattern of the subtropical climatic condition. The temperature pattern is characterized by hot summers and mild winters. The temperature variations may affect the performance of the PV system. The increase in temperature may decrease the efficiency of the PV system because of the increase in the internal resistance of the solar cells^49^.

#### Wind speed characteristics

**Fig. 5:**
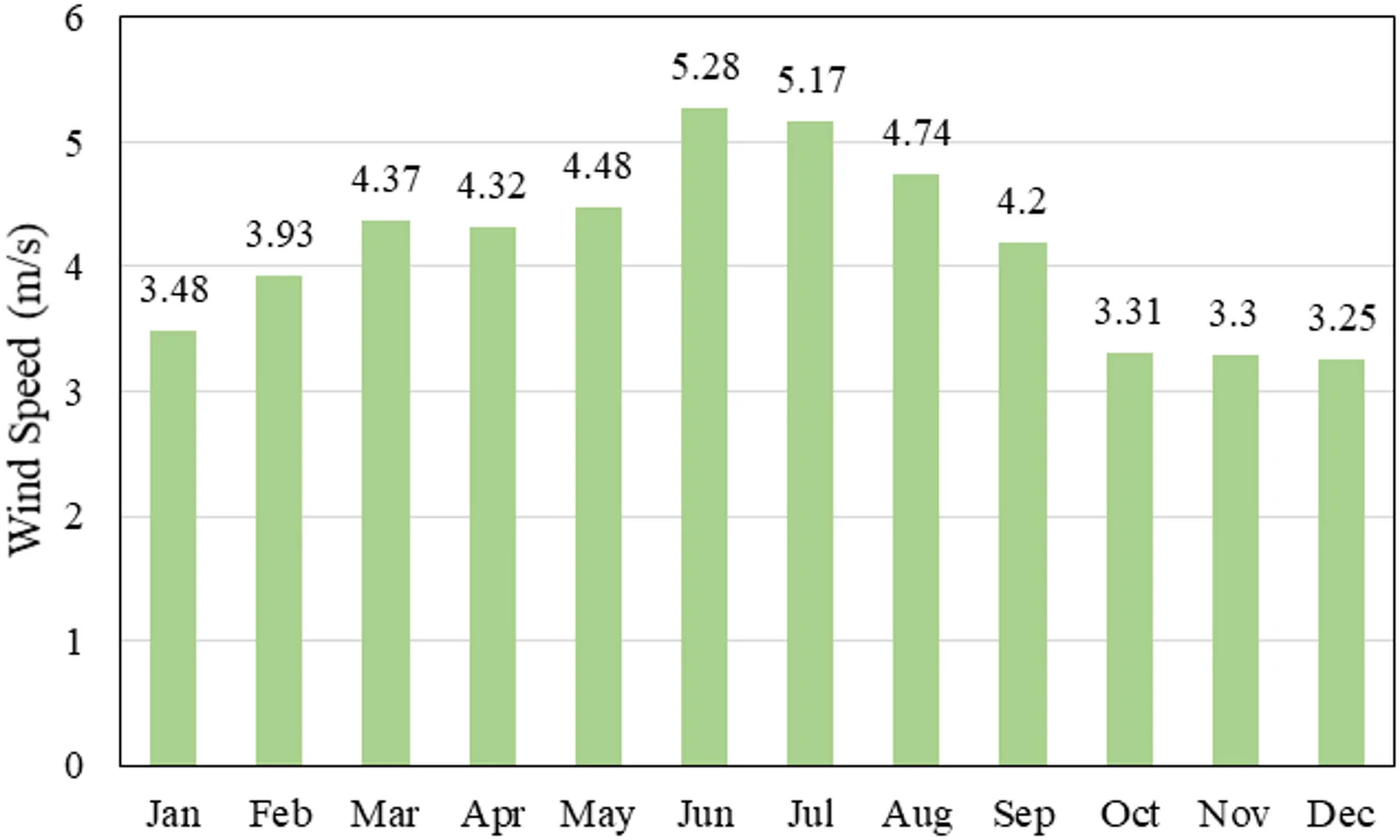
Seasonal variation in wind speed recorded at the study area.

Figure 5 shows the monthly mean wind speed progression. The annual cycle of wind speeds is at its lowest during the winter season. The lowest wind speed of 3.48 m/s is recorded in January and gradually increases to 3.93 m/s in February and further to 4.37 m/s in March. A moderate increase in the wind speed is also recorded during the spring season. The wind speed increases to 4.32 m/s in April and further to 4.48 m/s in May. The highest wind speeds are recorded between the months of June and July. During these two months of the year, the wind speed is at its peak. The speed is recorded at 5.28 m/s in June and further reduces to 5.17 m/s in July. Thereafter, the speed starts reducing and is recorded at 4.74 m/s in August and further reduces to 4.20 m/s in September. The lowest average wind speeds are recorded during the autumn and winter seasons. The speed is recorded at 3.31 m/s in October and further reduces to 3.30 m/s in November and to 3.25 m/s in December. A seasonal trend shows that summertime also comes with the best wind speeds for energy production. This phenomenon will influence planning because wind turbines operate best at higher wind speeds^50,51^.

### Biomass resource availability

**Fig. 6:**
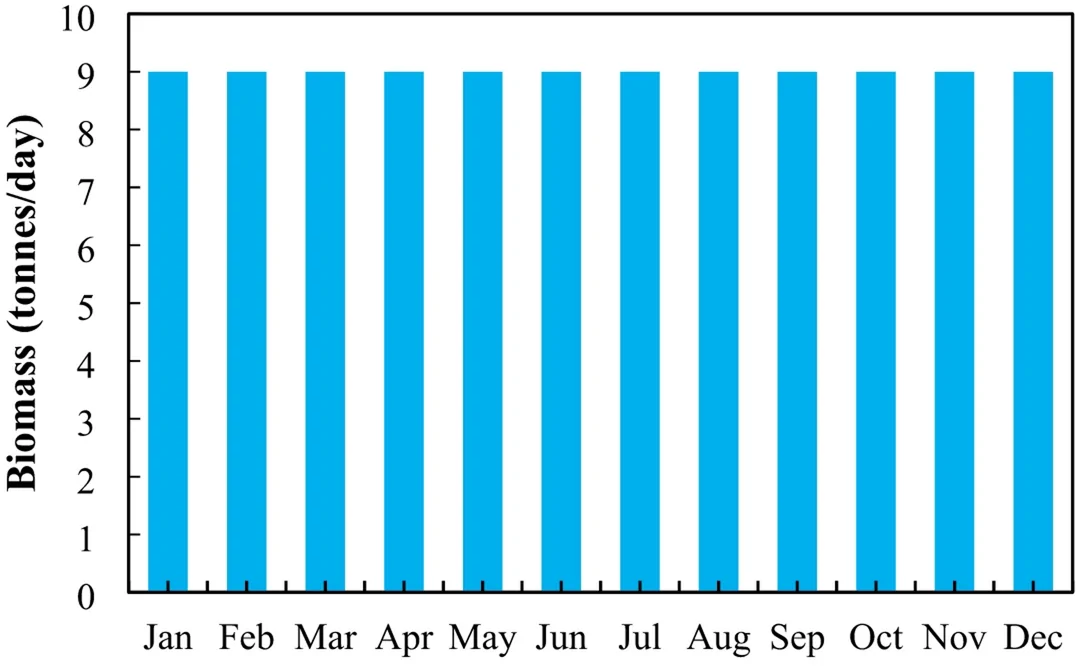
Estimated monthly availability of sustainable biomass resources at the site.

Figure 6 illustrates the monthly biomass available sustainably at the site of interest. It can be observed in the figure that there is a consistent yearly biomass supply, with an average of almost 9 tonnes/day from January to December. This indicates a consistent biomass supply throughout the year with minimal monthly variation. This is generally indicative of areas with consistent production of organic waste matter, including agricultural wastes, animal manure, and other biodegradable matter commonly found in rural areas. It indicates a consistent potential of biomass, which may be used as a potential renewable energy source in the production of biogas. It would provide a consistent power output in the community due to the consistent biomass supply in the area as a supplementary source of power in HRES systems, including solar and wind power.

### Microgrid architecture and simulation tool

**Fig. 7:**
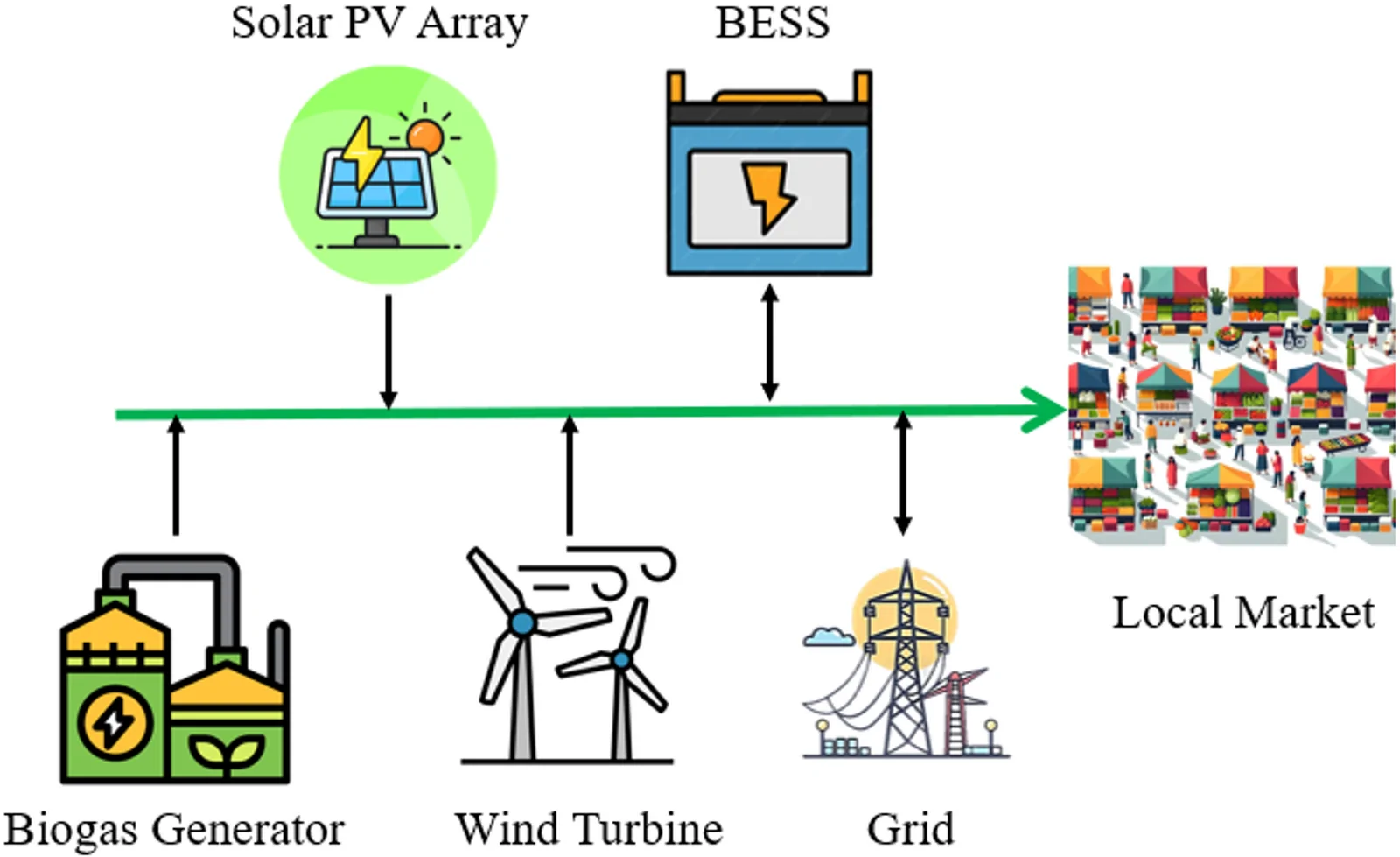
Proposed hybrid microgrid configuration for the Nazipur Noor market.

In that direction, therefore, the following microgrid has been architected to incorporate solar PV panels, a WT, a BioGen, and a BESS, with grid support for the Nazipur Noor market to handle electric load with variability in commercial demand. System modeling, simulation, and optimization were conducted through the use of software developed by the NREL, HOMER Pro v3.14.2^52^. It provides time-series simulation and techno-economic optimization of the systems under consideration based on NPC, COE, and renewable fraction as optimized key indicators. It is widely employed for the analysis of the performance of hybrid micro systems under different load and resource conditions^52,53^. The market demand is prioritized for the renewable generation; any surplus would either be stored in a BESS or sent to the grid, while any deficiency would be compensated with power from the grid ^54^. Optimization of the framework allows for an appreciation of reliable operation and realistic economic assessment over the project lifetime^55^. Figure 7, thus, shows the architected hybrid microgrid for integrate systems of PV arrays, WT, BioGen, BESS, and grid support, which was developed for the Nazipur Noor market. These sources are connected to a common bus that supplies the local market load. To maintain reliability, the BESS would be used until short-term fluctuations occur. The excess renewable energy will be stored or exported, and grid supply will fill the gap when the renewable generation is insufficient. The configuration allows flexible operations to adjust for the wide variation in commercial demand and resource conditions.

**Fig. 8:**
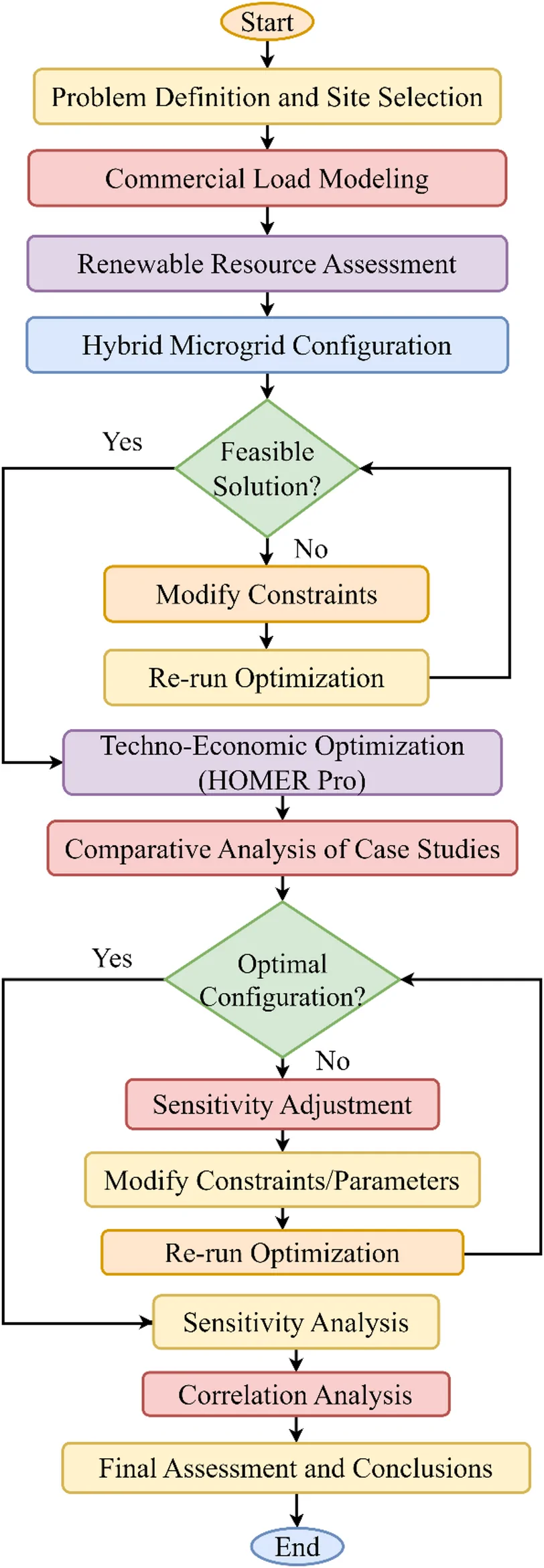
Integrated optimization and validation workflow for the proposed hybrid microgrid.

Figure 8 presents the integrated simulation, optimization, and validation workflow adopted in this study. The process begins with site selection and commercial load modeling, followed by renewable resource assessment and system configuration. Techno-economic optimization is performed using HOMER Pro v3.14.2, with iterative feasibility checks, comparative case analysis, and constraint refinement. Sensitivity and correlation analyses are conducted for optimal solutions prior to final performance assessment and conclusions.

HOMER Pro is selected in this study due to its robust capability for time-series simulation, techno-economic optimization, and sensitivity analysis of hybrid renewable energy systems. Compared to conventional optimization tools such as MATLAB-based algorithms, particle swarm optimization (PSO), or genetic algorithms (GA), HOMER Pro offers an integrated platform that simultaneously evaluates system feasibility, economic performance, and operational behavior under varying resource and load conditions. It enables rapid evaluation of a large number of system configurations with built-in models for renewable components, storage systems, and grid interaction. Furthermore, HOMER Pro incorporates real-world constraints such as resource variability, load fluctuations, and economic parameters, making it particularly suitable for practical microgrid planning applications. Unlike many analytical or metaheuristic optimization approaches that require external modeling and validation, HOMER Pro provides a validated and widely adopted framework specifically designed for hybrid energy system analysis, ensuring reliability and reproducibility of results.

#### Sensitivity and uncertainty analysis

Sensitivity analysis is conducted to evaluate the impact of key techno-economic and environmental parameters on the performance of the hybrid microgrid system. Critical variables such as wind speed, solar radiation, electricity tariff, and component costs are varied within predefined ranges to assess their influence on system configuration, cost of energy (COE), net present cost (NPC), and renewable fraction. HOMER Pro performs sensitivity analysis by simulating multiple scenarios under varying input conditions, allowing the identification of dominant parameters that significantly affect system feasibility and performance. In addition, uncertainty analysis is considered by examining variations in resource availability and economic parameters, providing a more robust evaluation of system reliability and long-term sustainability under real-world conditions.

The proposed microgrid system is formulated by integrating multiple energy sources, including solar PV, wind turbine, and biogas generator, along with battery energy storage and grid support. The system operates based on a priority control strategy in which renewable energy sources are utilized first to meet the load demand. Any excess energy is either stored in the battery or exported to the grid, while deficits are compensated through grid import. To evaluate system performance under different configurations, multiple scenarios are defined by varying the combination of generation sources and storage components. These configurations are analyzed under consistent load and resource conditions to identify the most cost-effective and sustainable system design. The detailed comparative results of these configurations are presented in Sect.  3.

### Modeling the components

Accurate component modeling is essential for evaluating the sizing and performance of a hybrid renewable energy system under site-specific conditions. This study adopts standard mathematical models, consistent with HOMER-based analysis, for all system components.

#### Solar PV

PV cells use solar radiation as a source of electrical power by converting it into electrical form through semiconductor materials like silicon. The electrical power generated by a PV system depends on various parameters like the rated capacity of the PV array, system derating factors, solar radiation, and temperature changes. These parameters are incorporated into the PV power output model presented in Eq. (1)^56,47^:

1 $$P_{{output}}^{{PV}} = P_{{rated}}^{{PV}} \times F_{D}^{{PV}} \times \left( {{\raise0.7ex\hbox{${G_{T} }$} \!\mathord{\left/ {\vphantom {{G_{T} } {G_{{T,STC}} }}}\right.\kern-\nulldelimiterspace} \!\lower0.7ex\hbox{${G_{{T,STC}} }$}}} \right) \times \left[ {1 + \eta _{P} \left( {T_{c} - T_{{c,STC}} } \right)} \right]$$

The PV output is constrained by the rated capacity of the system and inverter limitations, such that the generated power does not exceed the maximum allowable output. In practical simulation, the output is limited to the rated capacity whenever the calculated power exceeds this value.

2 $$P_{{PV}} \le P_{{rated~}}$$

This constraint ensures that the modeled PV output remains within physically realistic limits, accounting for practical system restrictions such as inverter capacity and module rating.

The term $\:{P}_{output}^{PV}$refers to the actual electrical power generated by the photovoltaic (PV) array (W), whereas $\:{P}_{rated}^{PV}$represents the rated capacity of the PV system under standard test conditions (W). The factor $\:{F}_{D}^{PV}$denotes the derating coefficient, which accounts for various system losses such as dust deposition, wiring inefficiencies, and other operational reductions. $\:{G}_{T}$indicates the solar irradiance incident on the surface of the PV modules (W/m²), while $\:{G}_{T,STC}$represents the irradiance level at standard test conditions, generally taken as 1000 W/m². The parameter $\:{\eta\:}_{P}$describes the temperature coefficient of power (%/°C), reflecting the sensitivity of PV output to temperature variations. In addition, $\:{T}_{c}$denotes the operating temperature of the PV cell (°C), whereas $\:{T}_{c,STC}$corresponds to the cell temperature under standard test conditions, which is typically assumed to be 25 °C.

#### Wind turbine

WT output power is modeled using the manufacturer’s power curve fitted by a least-squares quadratic function^57^:

3 $$\left\{ {\begin{array}{*{20}c} {{\mathrm{P}}_{{{\mathrm{WT}}}} \left( {\mathrm{t}} \right) = 0~;~~~~~~~~~~~~~~~~~~~~~~~~~~~~for~V < {\mathrm{V}}_{{{\mathrm{ci}}}} } \\ {{\mathrm{P}}_{{{\mathrm{WT}}}} \left( {\mathrm{t}} \right) = a{\mathrm{V}}^{2} + bV + c~;~~~~~~~~~~~~~for~{\mathrm{V}}_{{{\mathrm{ci}}}} < V < {\mathrm{V}}_{{\mathrm{r}}} } \\ {{\mathrm{P}}_{{{\mathrm{WT}}}} \left( {\mathrm{t}} \right) = {\mathrm{P}}_{{\mathrm{r}}} ~;~~~~~~~~~~~~~~~~~~~~~~~~~~for~V > {\mathrm{V}}_{{{\mathrm{co}}}} } \\ \end{array} } \right.$$

In this study, the wind turbine output between cut-in and rated wind speeds is approximated using a quadratic function derived from manufacturer power curve fitting (Eq. (3)). Although the theoretical wind power is proportional to the cube of wind speed, practical turbine output is often represented using polynomial approximations to reflect real operational behavior.

The rated power of a wind turbine can be expressed as:

4 $${\mathrm{P}}_{{\mathrm{r}}} = \frac{1}{2}{\mathrm{C}}_{{{p}}} {{~\rho }}_{{\mathrm{a}}} {{~\eta }}_{{{g}}} {\mathrm{A}}_{{\mathrm{w}}} {\mathrm{v}}_{{\mathrm{r}}}^{3} \:$$

For the WT operational model, $\:{P}_{WT}\left(t\right)$represents the electrical power generated by the wind turbine at time * t*, and * V*denotes the wind speed measured at the hub height. The parameters $\:{V}_{ci}$, $\:{V}_{r}$, and $\:{V}_{co}$correspond to the cut-in wind speed, rated wind speed, and cut-out wind speed, respectively. Additionally, the coefficients * a*, * b*, and * c*are quadratic fitting parameters used to approximate the wind turbine power curve within its operating range. The rated turbine power $\:{P}_{r}$depends on the power coefficient $\:{C}_{p}$, air density $\:{\rho\:}_{a}$, generator efficiency $\:{\eta\:}_{g}$, rotor swept area $\:{A}_{w}$, and rated wind speed $\:{V}_{r}$.

#### BioGen

The electrical energy output of the biogas generator is estimated based on the daily availability of biogas from cattle manure as^58^:

5 $${\mathrm{E}}_{{{\mathrm{BGG}}}} {{~}} = \frac{{{\mathrm{B}}_{{av}} \times {{~CV}}_{{{\mathrm{BGG}}}} {{~}} \times {{~\eta }}_{{{\mathrm{BGG}}}} {{~}} \times {{~}}\Delta {{t~}}}}{{860{{~}} \times {{~}}h_{{BGG}} }}$$

In the biogas generator model, $\:{E}_{BGG}$denotes the electrical energy output, where $\:{B}_{av}$is the available biomass feedstock, $\:C{V}_{BGG}$is the calorific value of biogas, $\:{\eta\:}_{IBGG}$is the electrical efficiency, $\:{\eta\:}_{BGG}$is the overall efficiency, and $\:{\Delta\:}t$is the operating time interval.

It is noted that the biogas generator output is initially expressed in terms of energy (kWh) over a given operating time interval. For consistency with the HOMER Pro simulation framework, this energy output is converted to equivalent power (kW) by dividing the generated energy by the corresponding operating duration. This formulation ensures consistency with other component models, which are represented in terms of instantaneous power output.

#### Converter

Converter losses are represented by a constant efficiency model^59^:

6 $$P_{{out}}^{{Con}} = P_{{in}}^{{Con}} \times \eta _{{Con}}$$

Converter performance is represented by $\:{P}_{in}^{Con}$and $\:{P}_{out}^{Con}$, which denote input and output power, respectively, with $\:{\eta\:}_{con}$indicating converter efficiency.

#### Battery storage

The SOC is updated dynamically as:

7 $$SOC\left( t \right) = SOC\left( {t - 1} \right) + \frac{{\eta _{{ch}} \cdot P_{{ch}} \left( t \right) \cdot {{\Delta }}t}}{{V_{{bus}} }} - \frac{{P_{{dis}} \left( t \right) \cdot {{\Delta }}t}}{{\eta _{{dis}} \cdot V_{{bus}} }}\:\:$$

where $\:SOC\left(t\right)$is the state of charge at time * t*, $\:{P}_{ch}\left(t\right)$and $\:{P}_{dis}\left(t\right)$represent the charging and discharging power, respectively, $\:{\eta\:}_{ch}$and $\:{\eta\:}_{dis}$are the charging and discharging efficiencies, $\:{\Delta\:}t$is the time step, and $\:{V}_{bus}$is the DC bus voltage. In Eq. (7), the first term represents the previous state of charge of the battery at time (t-1), reflecting the stored energy carried over from the preceding time step. The second term corresponds to energy accumulation during battery charging, while the third term accounts for energy reduction during battery discharging, with charging and discharging efficiencies appropriately considered. This formulation ensures an accurate representation of battery dynamics under varying operating conditions.

It should be noted that detailed dynamic battery aging mechanisms, such as cycle-dependent degradation and depth-of-discharge effects, were not explicitly modeled in this study. Instead, battery lifetime and replacement were represented using the standard HOMER Pro economic framework, which accounts for component replacement based on predefined operational lifetime assumptions. Future work may incorporate degradation-aware battery models for more detailed lifecycle assessment.

#### Utility grid

The annual grid energy cost is computed as^60^:

8 $$C_{{AEC}} = \mathop \sum \limits_{x}^{{{\mathrm{rates~}}}} \mathop \sum \limits_{y}^{{12}} E_{{gp,x,y}} P_{{{\mathrm{power~}},x}} - \mathop \sum \limits_{x}^{{{\mathrm{rates~}}}} \mathop \sum \limits_{y}^{{12}} E_{{gs,x,y}} P_{{{\mathrm{sellback~}},x}}$$

The annual grid demand charge is calculated by:

9 $$C_{{gd}} = \mathop \sum \limits_{x}^{{{\mathrm{rates~}}}} \mathop \sum \limits_{y}^{{12}} P_{{pgd,x,y}} D_{x} ~$$

For grid interaction, $\:{C}_{AEC}$represents the annual grid energy cost, where $\:{E}_{gp,x,y}$and $\:{E}_{gs,x,y}$denote grid-purchased and grid-sold energy, respectively, with corresponding electricity prices $\:{P}_{power,x}$and $\:{P}_{sellback,x}$. The annual grid demand charge is denoted by $\:{C}_{gd}$, where $\:{P}_{gpd,x,y}$is the grid peak demand and $\:{D}_{x}$is the demand charge rate.

### System modeling and economic evaluation

In order to ensure a comprehensive representation of the hybrid microgrid system, both technical and economic aspects are modeled using standard formulations consistent with HOMER Pro simulation principles. This subsection presents the governing equations for system operation, economic evaluation, and key parameters influencing system performance.

#### Power balance constraint

The operation of the hybrid microgrid is governed by the fundamental power balance equation:

10 $$P_{{PV}} \left( t \right) + P_{{WT}} \left( t \right) + P_{{{\mathrm{BioGen~}}}} \left( t \right) + P_{{{\mathrm{grid}}}} \left( t \right) = P_{{{\mathrm{load}}}} \left( t \right) + P_{{{\mathrm{battery}}}} \left( t \right) + P_{{{\mathrm{loss}}}} \left( t \right)$$

where $\:{P}_{PV}\left(t\right)$, $\:{P}_{WT}\left(t\right)$, and $\:{P}_{BioGen}\left(t\right)$represent the power generated from solar photovoltaic, wind turbine, and biogas generator, respectively, at time * t*. $\:{P}_{grid}\left(t\right)$denotes power exchanged with the utility grid (import/export), $\:{P}_{load}\left(t\right)$is the load demand, $\:{P}_{battery}\left(t\right)$represents battery charging/discharging power, and $\:{P}_{loss}\left(t\right)$accounts for system losses. This equation ensures that energy supply meets demand at all times under varying operating conditions.

#### Economic evaluation metrics

The economic performance of the system is evaluated using Net Present Cost (NPC), Cost of Energy (COE), and the Capital Recovery Factor (CRF).

The Net Present Cost is calculated as:

11 $$NPC = \frac{{C_{{ann}} }}{{CRF}}\:\:$$

where $\:{C}_{ann}$is the total annualized cost of the system. It should be noted that Eq. (11) assumes an equivalent uniform annualized cost $\:{C}_{ann}\:$over the project lifetime. This assumption is consistent with the HOMER Pro economic modeling framework, where annualized system costs are represented as constant equivalent values for net present cost (NPC) estimation.

The Capital Recovery Factor is defined as:

12 $$CRF = \frac{{i(1 + i)^{n} }}{{\left( {1 + i)^{n} - 1} \right.}}$$

where * i*is the annual discount rate and * n*is the project lifetime in years.

The Cost of Energy is expressed as:

13 $$COE = \frac{{C_{{ann}} }}{{E_{{served}} }}$$

where $\:{E}_{served}$represents the total electrical energy supplied to the load annually. These economic indicators provide a comprehensive assessment of system feasibility and long-term cost-effectiveness.

#### Reliability and operational parameters

Several system parameters, including hub height, grid failure frequency, mean repair time, variation in repair time, and discount rate, are incorporated into the simulation framework to reflect realistic operating conditions. Hub height directly influences wind speed and turbine output, while grid failure frequency and repair time parameters affect system reliability and grid interaction behavior. The discount rate plays a crucial role in determining the present value of future costs and revenues.

These parameters are implemented within HOMER Pro to simulate real-world uncertainties and operational constraints, thereby enabling a more accurate techno-economic evaluation of the hybrid microgrid system.

### Component specification and economic parameters

The system components and their respective capacities were chosen based on average technical specifications and economic conditions prevailing in the region of interest in South Asia. The capital cost, replacement cost, and O&M cost were based on data sheets and literature to obtain a realistic estimation of the costs associated with the system. As presented in Table 2, the system configuration under consideration consists of a 1 kW PV system, a 10 kW wind turbine, a 1 kW biogas generator, a 1 kW converter, and a 5.12 kWh battery energy storage system. These techno-economic data are critical to ensure the optimization and evaluation of various possible configurations of hybrid microgrid systems using HOMER Pro software.

It should be noted that the component sizes presented in the modeling section represent initial candidate values and search ranges used for optimization. The final system capacities reported in the Results section correspond to the optimal sizes determined by HOMER Pro based on techno-economic criteria. Therefore, differences in reported component sizes reflect the optimization outcome rather than inconsistencies in system design.

**Table 2:** Techno-economic parameters of system components.

Parameter	PV	WT	Converter	BioGen	BESS
Rated Capacity	1 kW	10 kW	1 kW	1 kW	5.12 kWh
Capital Cost ($)	300/kW	3000/unit	118/kW	85/kW	950
Replacement Cost ($)	300/kW	3000/unit	100/kW	70/kW	950
O&M Cost ($/yr)	10/kW	50/unit	10/kW	0.07/kW	1
Reference	^61^	^62^	^63^	^64^	^65^

The component sizes listed in Table 2 are selected based on values reported in previous studies and manufacturer data. These values define the feasible search space for the optimization process rather than fixed system design parameters. HOMER Pro evaluates different combinations within these ranges to determine the optimal system configuration based on techno-economic criteria. The minimum values (e.g., 1 kW for PV) are chosen to enable scalable system design and to allow the optimization algorithm to explore a wide range of feasible configurations with sufficient resolution. The selected parameter ranges are based on reported values in the literature and are used to define the optimization search space. The converter enables bidirectional power flow between AC and DC subsystems, ensuring proper integration of renewable sources and storage with the load demand.

To evaluate the performance of different system configurations, a total of eight case studies are considered in this work, including one base case and seven hybrid configurations combining PV, wind turbine, biogas generator, battery storage, and grid support. In addition, sensitivity analysis is performed to assess the influence of key techno-economic parameters such as wind speed, solar radiation, and electricity pricing. The detailed results and comparative analysis of these cases are presented in Sect.  3.

## Results and discussion

Based on the case study configurations defined in Sect.  2, a total of eight system scenarios are evaluated using HOMER Pro. The results are analyzed in terms of techno-economic performance, renewable energy penetration, and environmental impact. In total, 1,056 system configurations were simulated, reflecting different combinations of PV, wind turbine, biogas generator, battery storage, converter, and grid interaction under varying techno-economic conditions. Among these, 829 configurations satisfied the feasibility criteria, while 227 cases were rejected due to capacity shortage constraints. In addition, 232 configurations were excluded due to technical infeasibility or inappropriate converter selection, ensuring that only practically viable solutions were retained for further analysis. The total number of simulated configurations was obtained from the combinations of predefined search ranges assigned to PV capacity (0–150 kW), wind turbine units (0–3), and biogas generator capacity (0–150 kW) across the investigated case studies. Battery energy storage system (BESS) capacity and converter sizing were automatically optimized within HOMER Pro to ensure economically feasible and technically reliable solutions. The corresponding search ranges and component combinations considered in each case study are summarized in Table 3.

As summarized in Table 3 the investigated case studies include eight predefined system configurations based on component inclusion, including a base case and seven hybrid scenarios. These cases represent distinct system architectures used for comparative analysis, whereas the total number of simulated configurations (1,056) arises from variations in component sizing and sensitivity parameters within each case. This structured classification enables a systematic comparison of techno-economic performance, renewable penetration, grid dependency, and environmental impact across different system architectures, providing a clear basis for identifying the most suitable configuration for market-level electrification.

**Table 3:** Overview of investigated case studies and optimization input search ranges.

Case Study	Components Included	PV (kW)	WT	BioGen (kW)	BESS	Converter
Base Case	BESS–Grid–Converter	0	0	0	HOMER optimizer	HOMER optimizer
Case-I	PV–WT–BioGen–BESS–Grid–Converter	0–150	0–3	0–150	HOMER optimizer	HOMER optimizer
Case-II	PV–BioGen–BESS–Grid–Converter	0–150	0	0–150	HOMER optimizer	HOMER optimizer
Case-III	PV–BESS–Grid–Converter	0–150	0	0	HOMER optimizer	HOMER optimizer
Case-IV	WT–BioGen–BESS–Grid–Converter	0	0–3	0–150	HOMER optimizer	HOMER optimizer
Case-V	WT–BESS–Grid–Converter	0	0–3	0	HOMER optimizer	HOMER optimizer
Case-VI	PV–WT–BESS–Grid–Converter	0–150	0–3	0	HOMER optimizer	HOMER optimizer
Case-VII	BioGen–BESS–Grid–Converter	0	0	0–150	HOMER optimizer	HOMER optimizer

### Techno-economic analysis

The techno-economic analysis compares the feasibility, cost-effectiveness, and sustainability of different hybrid microgrid configurations under the same technical and operational conditions. As shown in Table 4, the Base Case exhibits the highest NPC and COE at USD 433,497.1 and USD 0.149/kWh, respectively, due to its complete dependence on grid electricity without renewable energy integration. In contrast, hybrid configurations incorporating renewable energy sources demonstrate significantly improved techno-economic performance. Among all cases, Case I achieves the lowest NPC and COE at USD 91,255.5 and USD 0.0167/kWh, respectively, along with a high renewable fraction of 90.97%, indicating superior system performance.

It is important to note that each configuration is independently optimized in HOMER Pro, and therefore the component capacities (e.g., PV, wind turbine, biogas generator, battery storage, and converter) vary across different cases to achieve the optimal techno-economic performance. As a result, the system cost is not solely determined by the inclusion or exclusion of specific components. For instance, configurations without battery storage may require higher generation capacity or increased grid dependency to meet load demand and reliability constraints, which can lead to higher overall cost despite having fewer components. Overall, the results highlight the trade-off between capital investment, operating cost, and renewable energy penetration in determining the most suitable configuration for sustainable market electrification.

**Table 4:** Comparison of techno-economic and environmental performance indicators for the studied hybrid microgrid configurations.

Case Study	NPC ($)	COE ($)	Operating cost ($/yr)	Initial capital ($)	Ren Frac (%)	Energy Purchased (kWh)	Energy Sold (kWh)	CO_2_ & CO (kg/yr)	SO_2_ & NO_x_ (kg/yr)
Base Case	433497.1	0.149	29787.35	34110.37	0	219455.7	110.6584	136,063 & 0	601 & 294
Case-I	91255.5	0.0167	391.78	86002.55	90.97	36723.33	189020.1	22,516 & 0.444	99.3 & 48.9
Case-II	184379.3	0.0348	7863.99	78939.43	86.00	55282.65	177253.2	34,357 & 0.907	151 & 74.6
Case-III	185835.1	0.0473	8311.496	74395.16	65.43	101255.3	75513.7	62,778 & 0	277 & 136
Case-IV	191110.2	0.0401	11186.66	41120.29	78.00	78142.84	137702.3	48,540 & 1.01	214 & 105
Case-V	252134.7	0.0791	16097.43	36301.5	46.01	128353.7	20244.21	79,579 & 0	352 & 172
Case-VI	279682.5	0.0657	7340.152	181266.3	95.44	14499.1	100263.6	8,989 & 0	39.7 & 19.4
Case-VII	305607.3	0.0652	21487.7	17501.74	64.57	123,811	131854.8	76,899 & 1.51	339 & 167

**Fig. 9:**
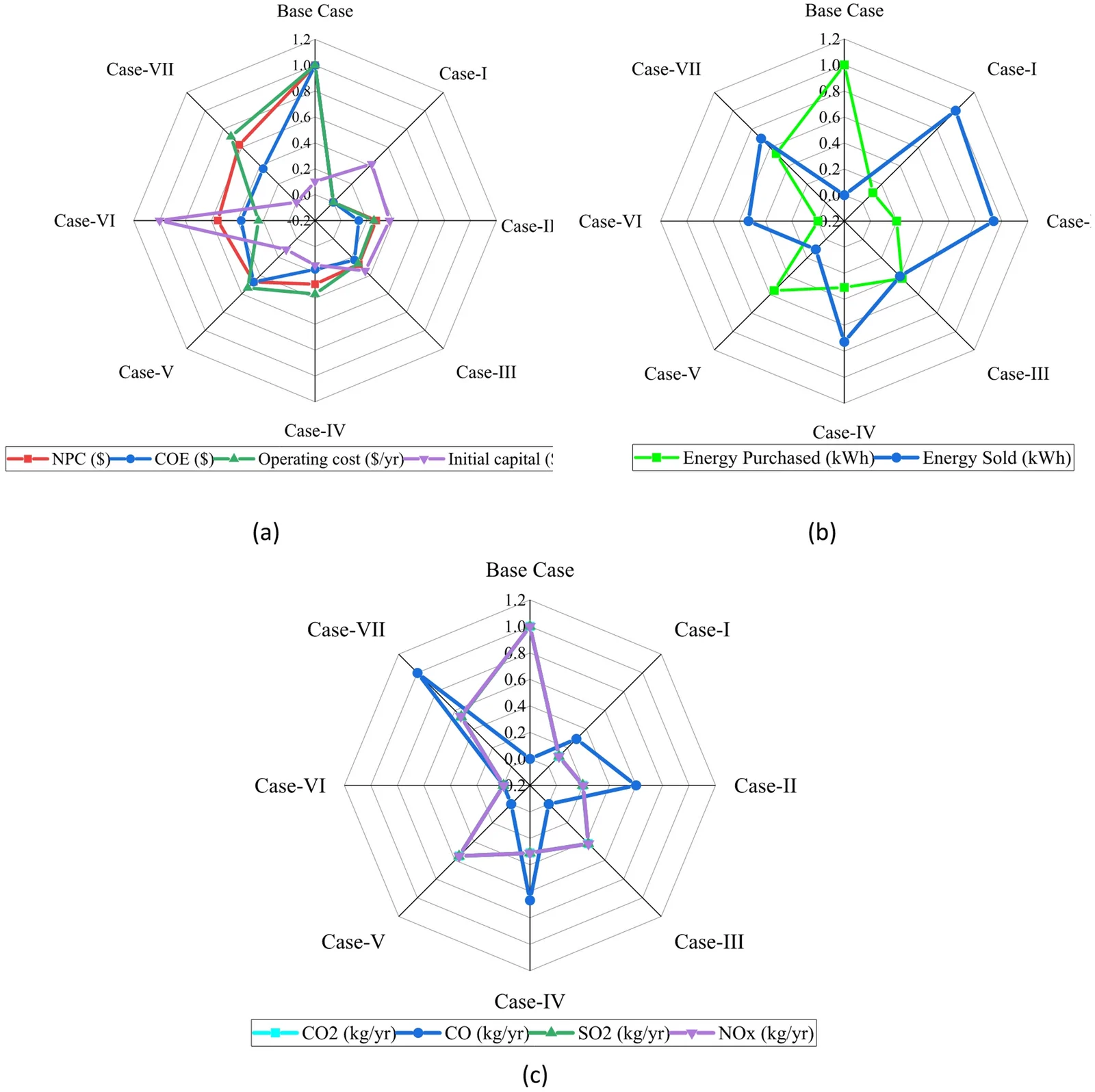
Comparative spider plots of normalized (**a**) techno-economic indicators, (**b**) grid energy exchange, and (**c**) emission metrics for all case studies.

Figure 9 presents the comparison of the evaluated hybrid configurations using normalized spider plots. Figure 9(a) below presents the normalized techno-economic indicators NPC, COE, Operating Cost, and Initial Capital in the range of 0 to 1. This will allow the comparison of the results of all case studies. The Base Case exhibits the worst overall performance, with all major cost indicators normalized to 1.0, reflecting the highest NPC (USD 433,497.1), COE (USD 0.149/kWh), and operating cost (USD 29,787.35/yr). Case-I demonstrates the best techno-economic performance, with normalized values of 0 for NPC, COE, and operating cost, despite a higher initial capital (normalized ≈ 0.418). Case-II to Case-IV show moderate performance, with normalized NPC values between 0.272 and 0.292 and operating cost between 0.254 and 0.367. Case-VI exhibits a high normalized initial capital (1.0) due to its large investment, while Case-VII shows relatively high normalized operating cost (0.718), indicating weaker long-term economic performance. Figure 9(b) compares energy purchased and energy sold, normalized to highlight grid interaction behavior. The Base Case shows complete grid dependence, purchasing 219,455.7 kWh (normalized = 1.0) and selling only 110.66 kWh (normalized = 0). Case-I achieves the strongest export performance, selling 189,020.1 kWh (normalized = 1.0) while purchasing only 36,723.33 kWh (normalized ≈ 0.108). Case-II also performs strongly, selling 177,253.2 kWh (normalized ≈ 0.938) with moderate imports. Case-VI shows the lowest grid purchase (14,499.1 kWh; normalized = 0) but also lower exports compared to Case-I. Case-VII exhibits moderate grid purchase (123,811 kWh; normalized ≈ 0.533) with relatively limited export. Figure 9(c) illustrates normalized emission indicators (CO₂, CO, SO₂, NOₓ). The Base Case again represents the worst environmental performance, with CO₂ = 136,063 kg/yr, SO₂ = 601 kg/yr, and NOₓ = 294 kg/yr, all normalized to 1.0. Case-VI shows the best environmental outcome, with the lowest emissions (CO₂ = 8,989 kg/yr; SO₂ = 39.7 kg/yr; NOₓ = 19.4 kg/yr; all normalized ≈ 0). Case-I also demonstrates strong emission reduction (CO₂ = 22,516 kg/yr; normalized ≈ 0.109). In contrast, Case-VII exhibits higher normalized CO and NOₓ values, indicating weaker emission performance. Overall, the radar plots clearly highlight Case-I as the best techno-economic and grid-interactive option, while Case-VI excels environmentally, and the Base Case remains the least sustainable across all dimensions.

**Fig. 10:**
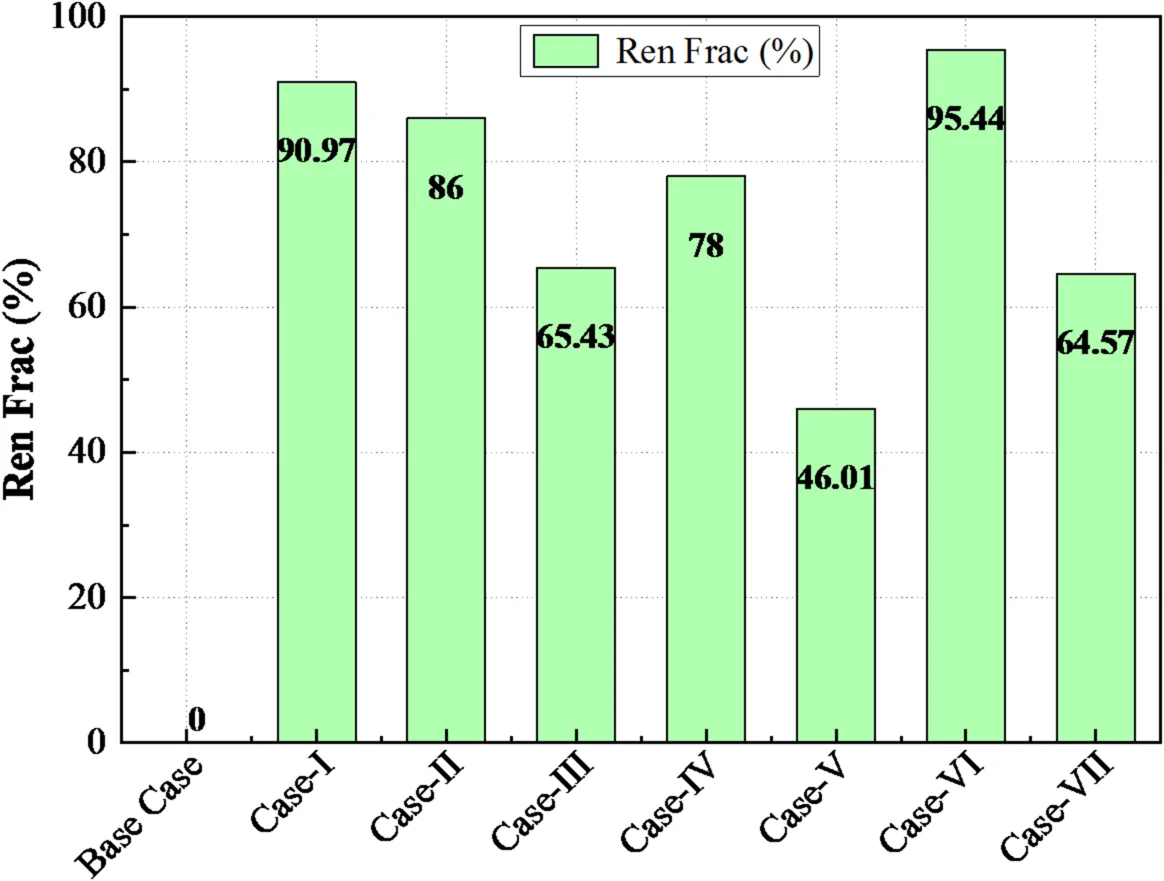
Contribution of renewable energy sources under the optimized microgrid scenarios.

The renewable energy fraction for all case studies is presented in Fig. 10 below. For the case studies, the Base Case results in 0%, meaning that it is totally dependent on grid power. From the results, Case-VI results in the highest percentage, 95.44%, followed by Case-I, 90.97%, and Case-II, 86%. Case-IV attains a renewable fraction of 78%, while Case-III and Case-VII show moderate contributions of 65.43% and 64.57%, respectively. Case-V records the lowest renewable penetration among hybrid systems at 46.01%, reflecting higher grid reliance.

The use of a realistic market-based load profile significantly influences system design and performance outcomes. Compared to simplified or static load assumptions, the proposed approach results in higher peak demand estimation and improved representation of daytime load concentration. This leads to more accurate sizing of system components, particularly battery storage and generation capacity. As a result, the optimized system achieves a more reliable balance between renewable generation and grid interaction, reducing the risk of underestimation of system requirements observed in conventional approaches.

### Optimum case

The primary finding of this study is that the PV–WT–BioGen–BESS–Grid configuration (Case I) represents the optimal hybrid microgrid solution for the considered market load, achieving the best balance between economic performance, renewable energy penetration, and environmental sustainability. Among all evaluated configurations, Case I is identified as the optimal solution based on its lowest NPC and COE, highest renewable fraction, and superior environmental performance. This microgrid configuration has achieved the lowest NPC value of USD 91,255.5 and the lowest cost of electricity of USD 0.0167/kWh. These figures demonstrate excellent long-term economic efficiency. Additionally, Case I has achieved the lowest operating cost of USD 391.78 annually among all the renewable-rich microgrid configurations. This is a clear indication that Case I has minimized its dependence on grid electricity and has maximized its use of renewable resources. Although Case I has a high initial capital cost compared to some other microgrid configurations, its ability to minimize its operating costs outweighs its initial capital cost, making Case I the optimum solution.

**Fig. 11:**
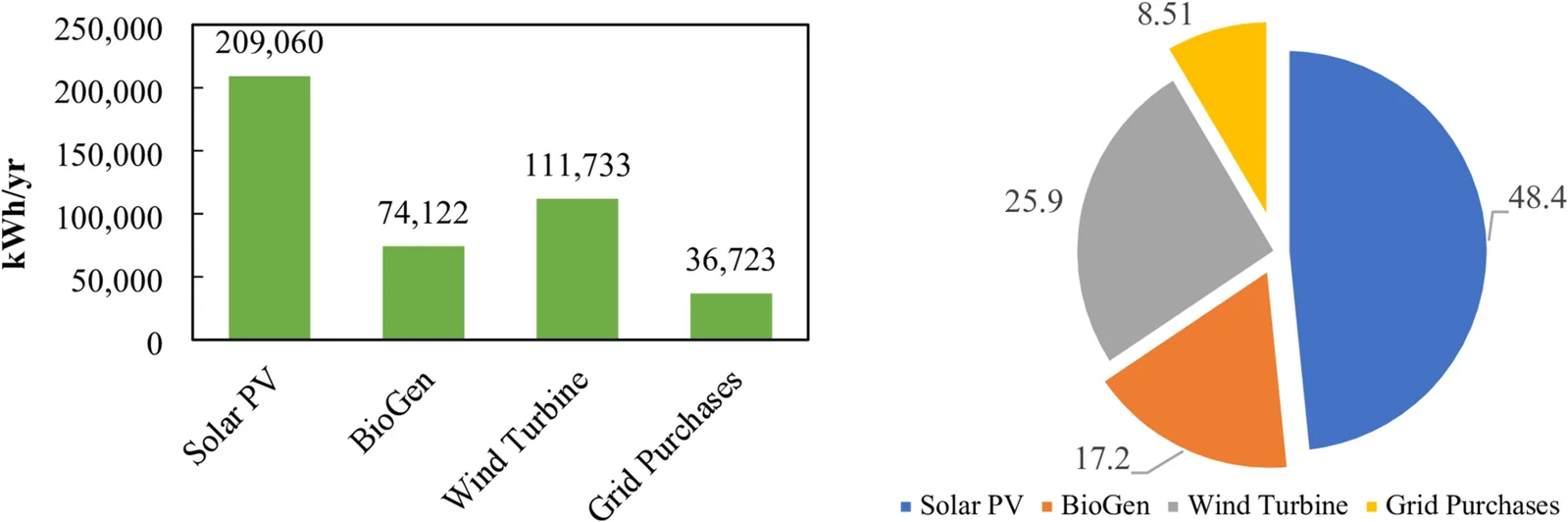
Annual energy generation and percentage contribution of each source in the optimal hybrid system.

Figure 11 below shows the optimum hybrid microgrid solution’s annual energy contribution and source. Solar is the dominant energy source, making 48.4%, or 209,060 kWh/year, followed by the wind turbine, making 25.9%, or 111,733 kWh/year, and the biogas generator making 17.2%, or 74,122 kWh/year. Grid electricity purchases account for only 36,723 kWh/year (8.51%), demonstrating limited grid dependence and strong renewable penetration.

Consequently, Case I achieves a high renewable fraction, confirming its robust integration of PV–WT–BioGen–BESS components and its superior sustainability performance compared with the other configurations.

**Fig. 12:**
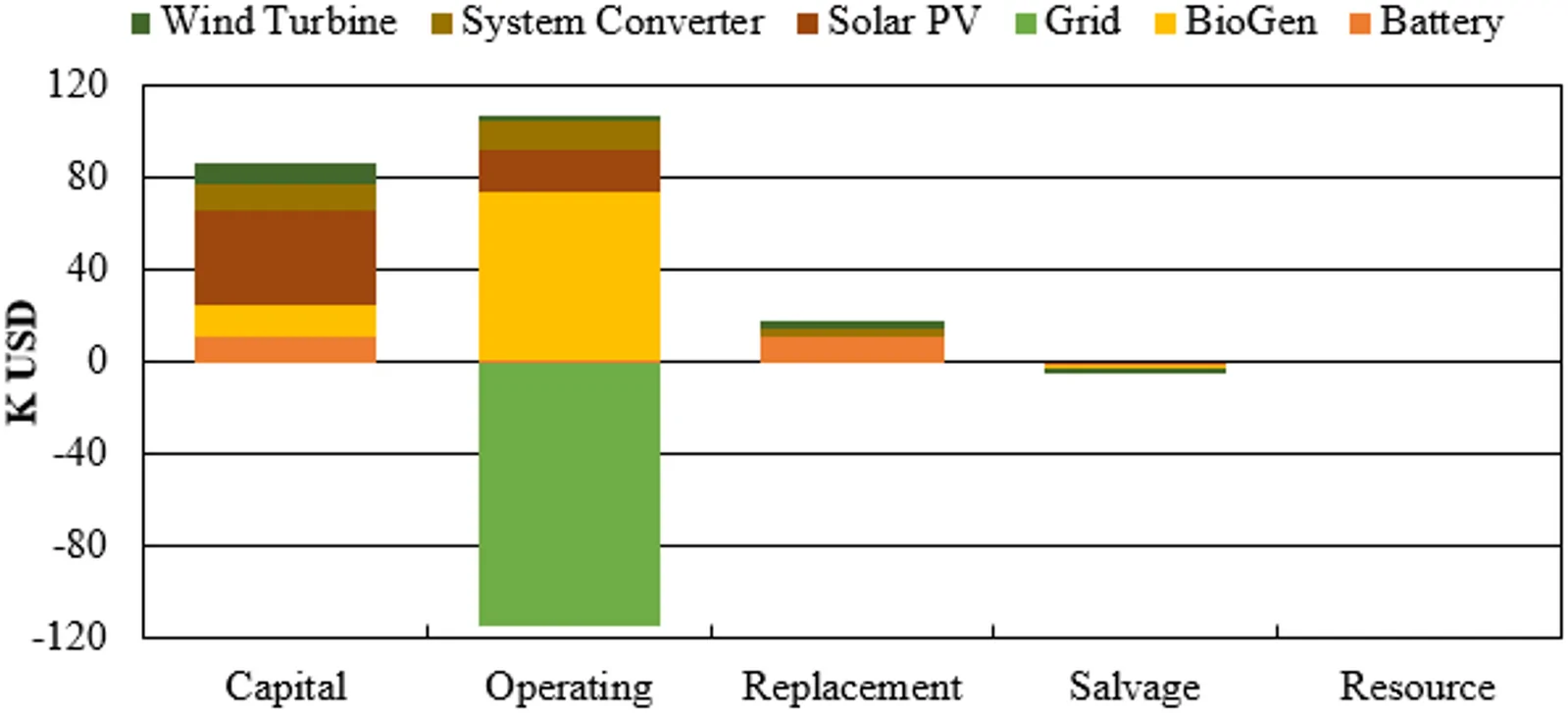
NPC contribution of individual components in the optimal system configuration.

Figure 12 illustrates the contribution of individual system components to the NPC of the optimal Case I configuration. Solar PV represents the largest positive cost contributor, with a total NPC of USD 65,112, primarily driven by its capital cost of USD 45,000 and operating cost of USD 20,112. The biogas generator contributes USD 84,995, including USD 12,750 in capital cost and USD 73,207 in operating cost. In contrast, the grid shows a negative NPC of − USD 119,486, reflecting revenue from electricity export. The battery system (LiFePO₄ 51.2 V) contributes USD 20,643, while the wind turbine and converter contribute USD 12,325 and USD 27,016, respectively. Overall, grid revenue significantly offsets component costs, resulting in a total system NPC of approximately USD 90,604.

**Fig. 13:**
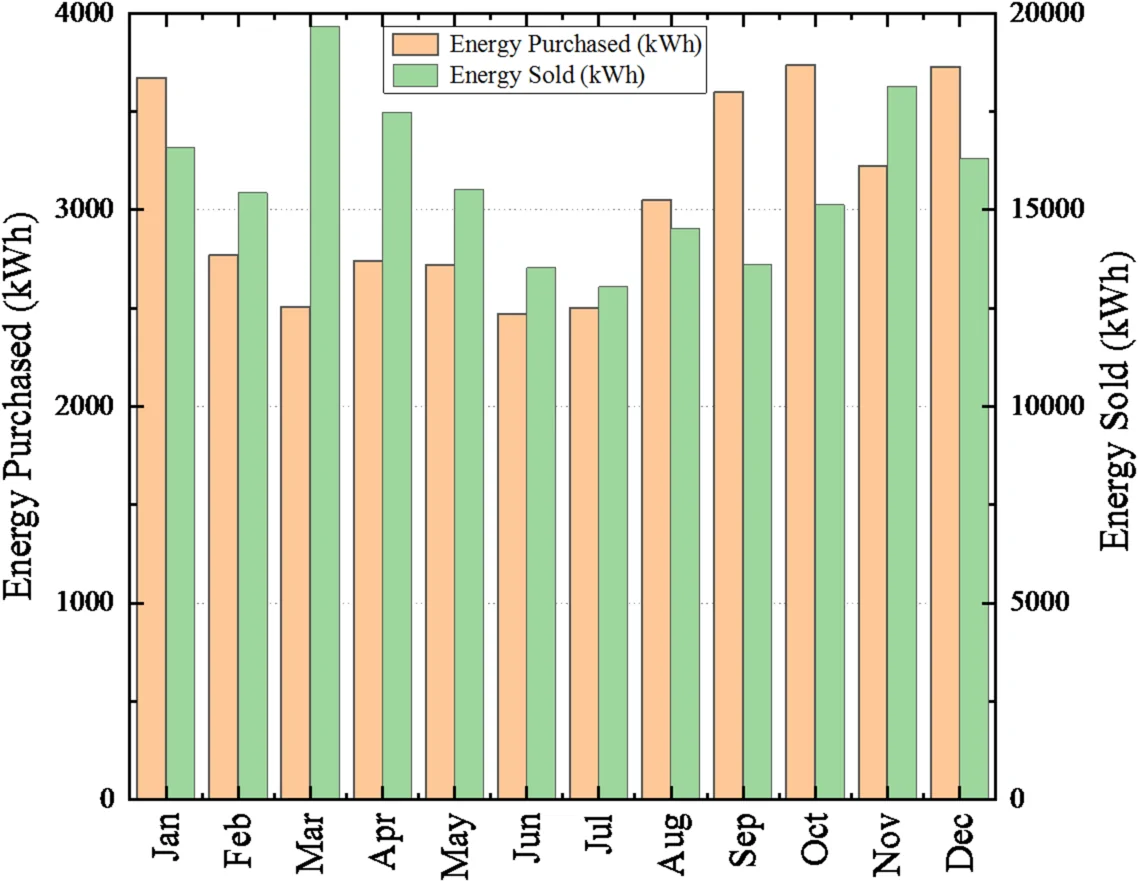
Monthly energy purchased and sold for the optimal Case I configuration.

As shown in Fig. 13, the monthly grid energy exchange in the optimal configuration with Grid Rate 1: The amount of energy purchased from the grid remains stable throughout the year, with a range of 2,425 kWh in June and 3,683 kWh in December. Annual total: 36,723.33 kWh. In contrast, excess electricity is exported to the utility grid is significantly higher in all months, varying between 13,440 kWh (July) and 20,258 kWh (March), resulting in an annual export of 189,020.1 kWh. In every month, exported energy exceeds grid purchases, indicating consistent surplus renewable generation. This pattern confirms strong grid-interactive operation with reduced grid dependence and effective utilization of excess renewable energy.

**Fig. 14:**
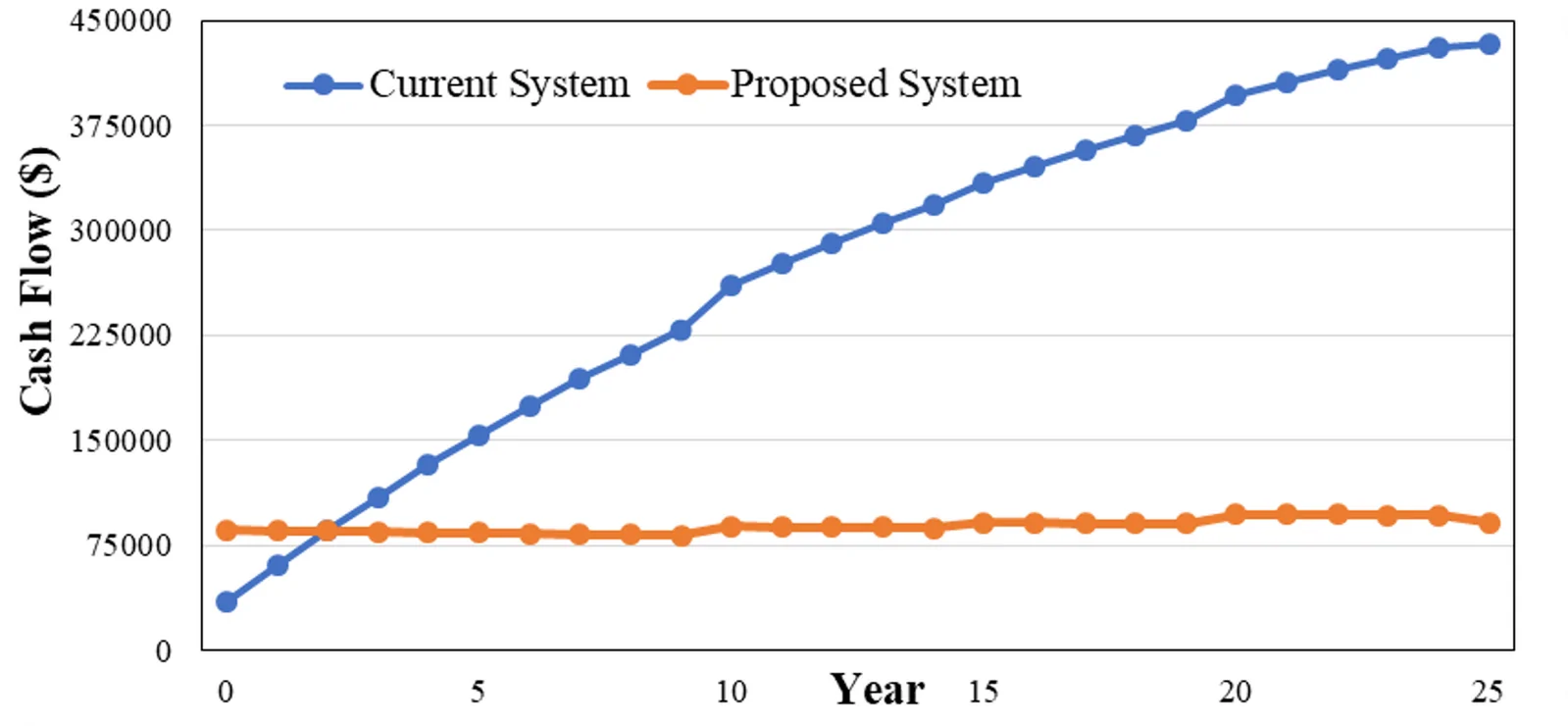
Cumulative cash flow over project lifetime.

Figure 14 illustrates the cumulative cash flow comparison between the current system and the proposed hybrid microgrid system for the project period. From the figure, it is clear that the proposed system has a capital investment of USD 55,104, reduces the operating expenses by USD 103.63 annually, and thus has an annual savings of USD 29,684 for the proposed system. The simple payback period for the proposed system is 1.91 years, and the IRR is high at 52.6%, with a return on investment of 50%. From the figure, it is also clear that the cumulative cash flow for the proposed system is always less compared to the current system, with a NPV of USD 342,893.

**Fig. 15:**
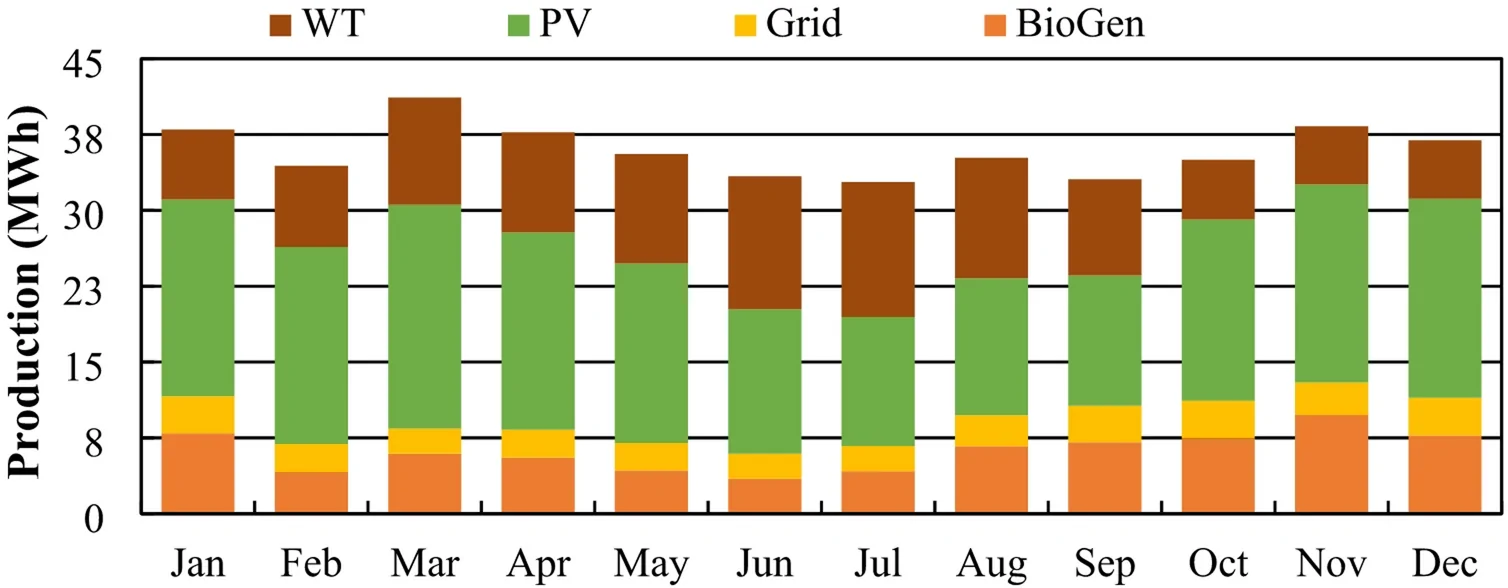
Monthly electricity generation from renewable sources and the grid (MWh).

Figure 15 below shows the monthly electricity generated by the proposed microgrid. It is clear that renewable energy sources dominate the microgrid power generation throughout the year, effectively meeting the market electricity demand under varying operating conditions.

**Fig. 16:**
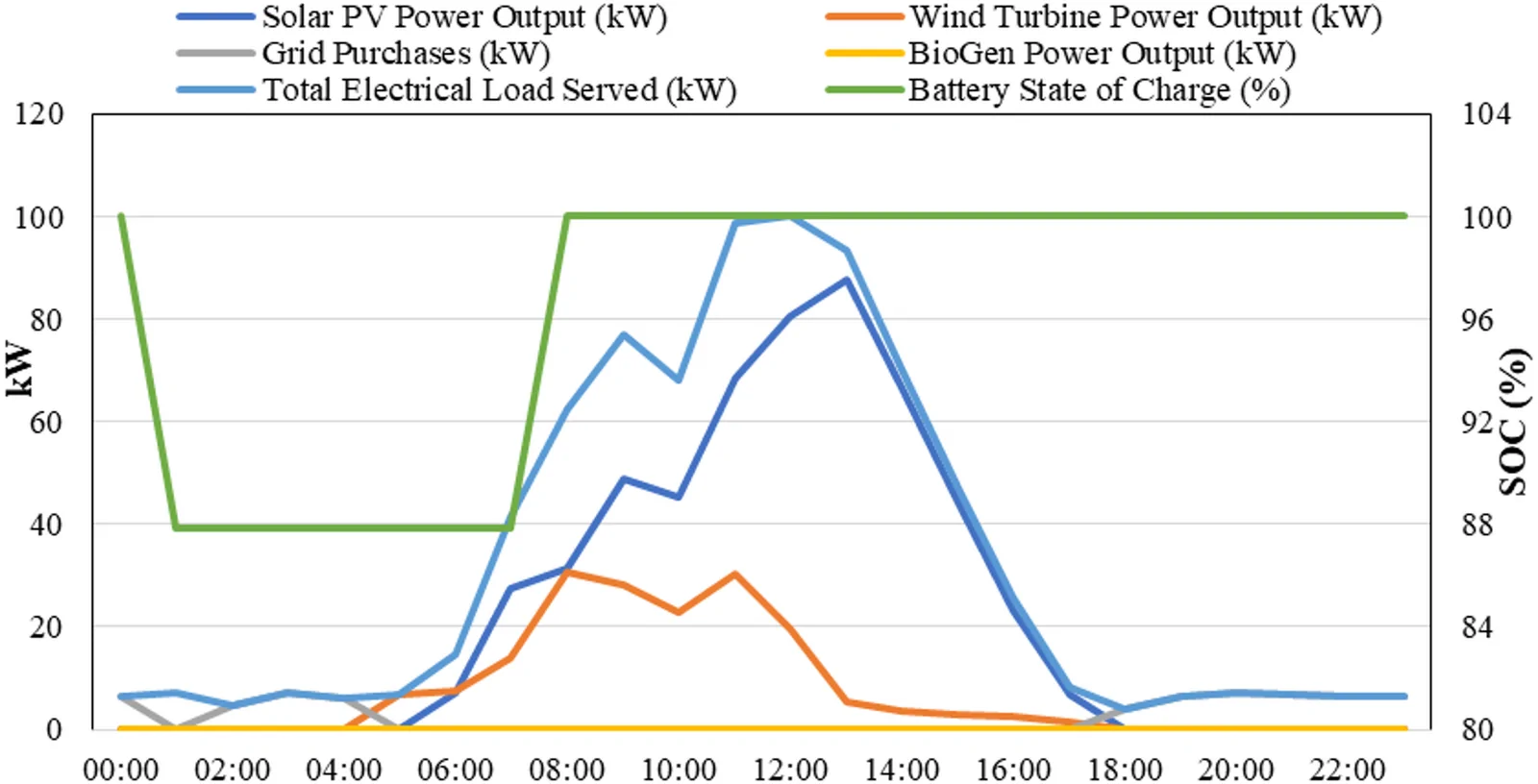
Hourly power dispatch pattern and battery SOC profile on 1 April.

Figure 16 shows the hourly power dispatch and SOC on 1 April. During the night and early morning (00:00–05:00), solar PV output is nearly zero, wind output remains low (generally below ~ 10 kW), and the load is supported by a combination of grid import and battery discharge, causing the battery SOC to drop from about 100% to approximately 88%. After sunrise (≈ 06:00), PV generation increases rapidly, reaching a peak of around 95–100 kW near midday, while wind contributes up to ~ 25–30 kW. This surplus generation supplies the load and recharges the battery to ~ 100% SOC. In the afternoon, as PV output declines, SOC remains stable due to reduced load and sufficient renewable support. Overall, the battery operates within a narrow SOC range, indicating that it primarily functions as a backup and balancing unit rather than undergoing deep discharge cycles.

On the whole, Case I proves to be the best option with the least NPC ($91,255.5) and COE ($0.0167), along with the lowest operating cost and maximum renewable penetration (90.97%). It significantly reduces grid reliance and emissions with a reliable operation, hence proving the suitability of Case I for microgrid development.

**Fig. 17:**
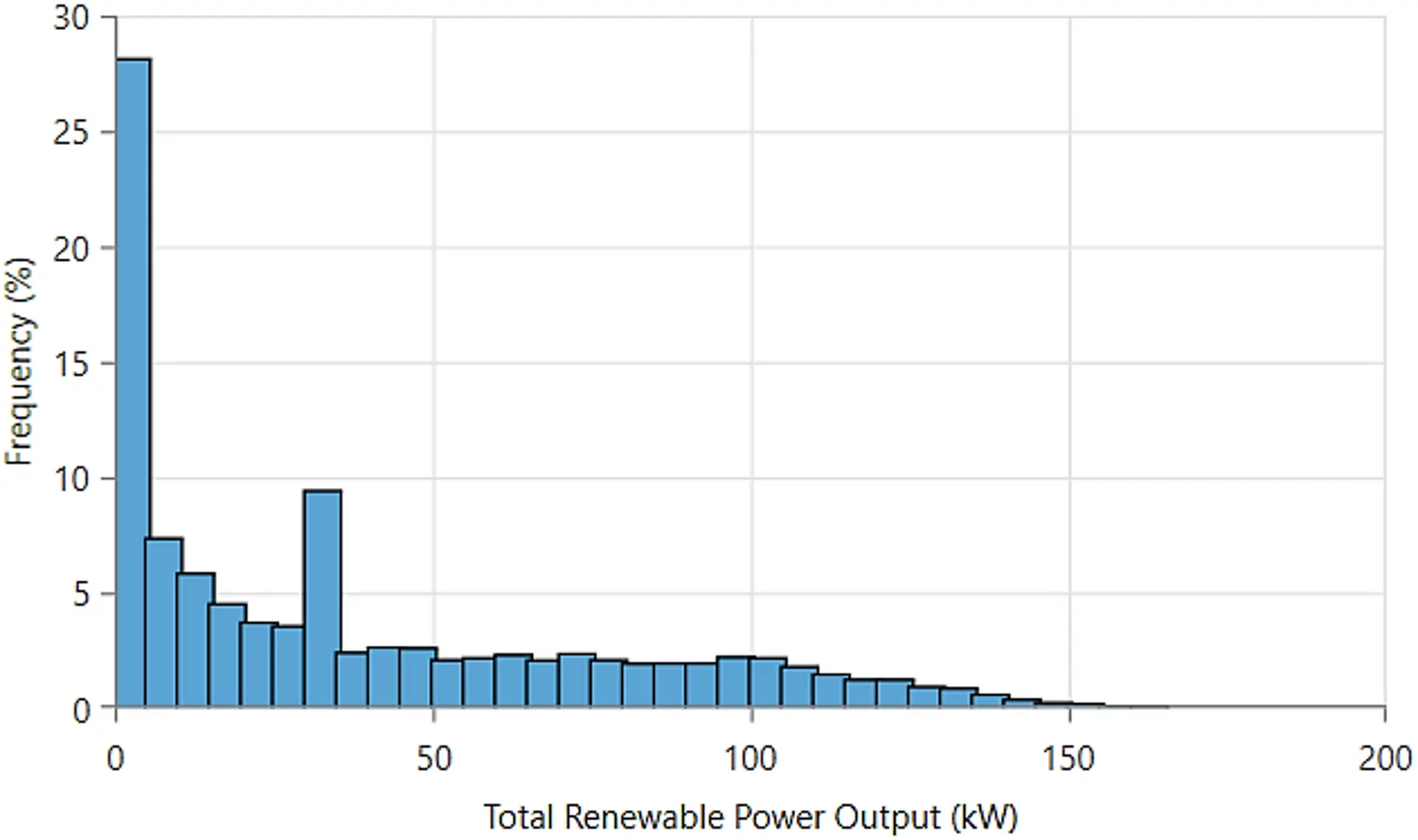
Histogram illustrating the distribution of total renewable energy generation (kW).

Figure 17 shows the histogram of the total renewable power output of the proposed microgrid system. It is observed that the histogram is highly skewed to lower values of renewable power outputs. This indicates that the microgrid system operated at lower renewable power outputs for most of the hours of operation. A significant number of hours of operation were observed to have renewable power outputs lower than 20 kW, which may be due to low solar irradiance and wind speed. However, renewable power outputs between 20 kW and 60 kW were observed for moderate hours of operation, whereas outputs greater than 100 kW were observed for fewer hours of operation. When renewable power outputs increased from 120 kW, it was observed that their frequency of occurrence decreased significantly, whereas very high renewable power outputs near 150–160 kW were observed for fewer hours of operation.

**Fig. 18:**
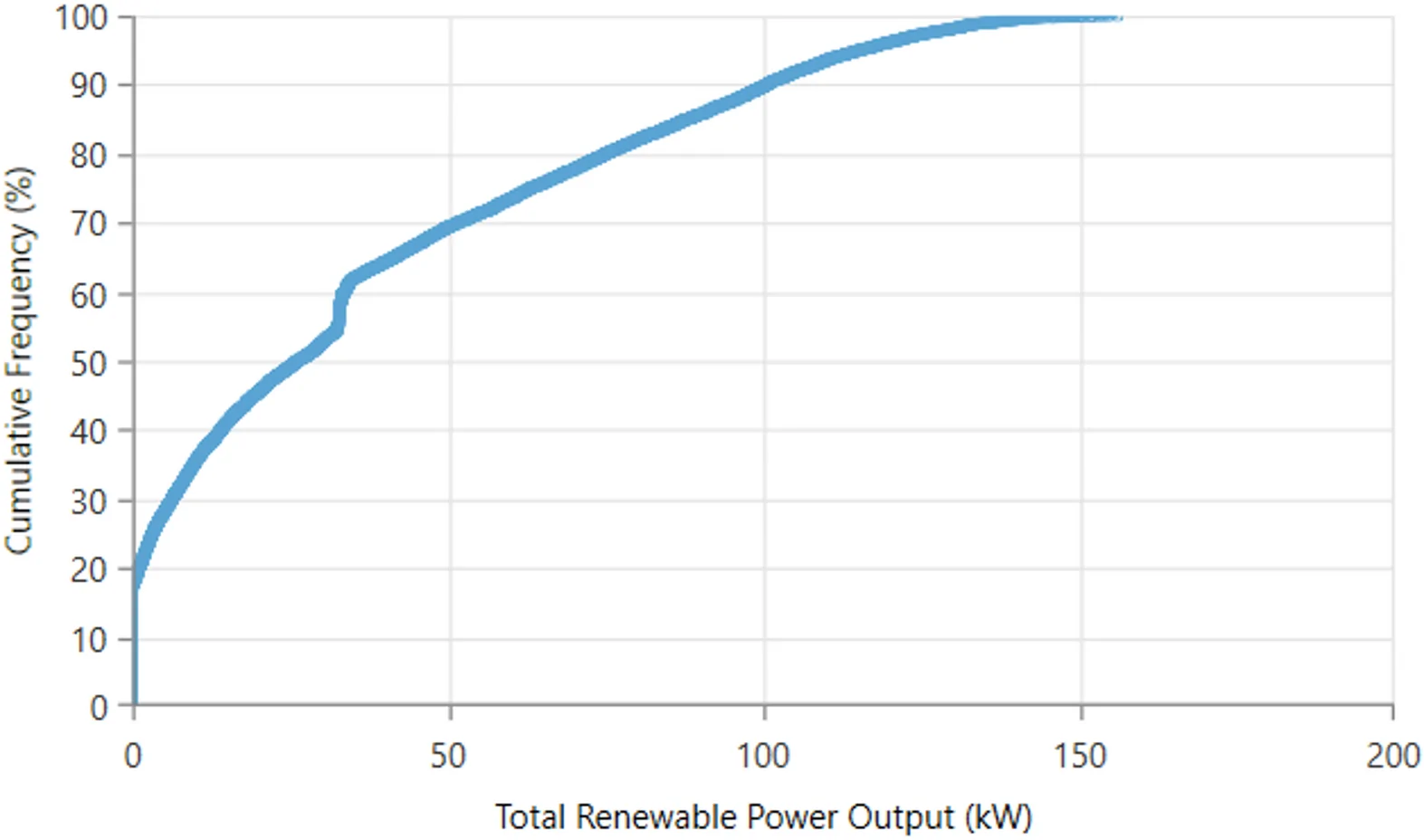
Cumulative distribution function (CDF) representing the overall renewable power output.

As shown in Fig. 18, the graph of the cumulative distribution function (CDF) of the total power output of renewable resources indicates that on a particular occasion, nearly 50% of the time, the power output of renewable resources was below 40 kW. It also indicates that nearly 70% of the total time was below 60 kW, and nearly 90% of the total operating time was below 120 kW. However, only a small percentage of the total time was near the peak power output of the system, which was nearly 150–160 kW. This indicates the dominance of low power output levels. Further, the steep curve of the CDF at low power output levels indicates the variability of solar and wind resources, whereas the flat curve of the CDF at the peak power output level indicates that the peak power output of renewable resources was rarely reached.

**Fig. 19:**
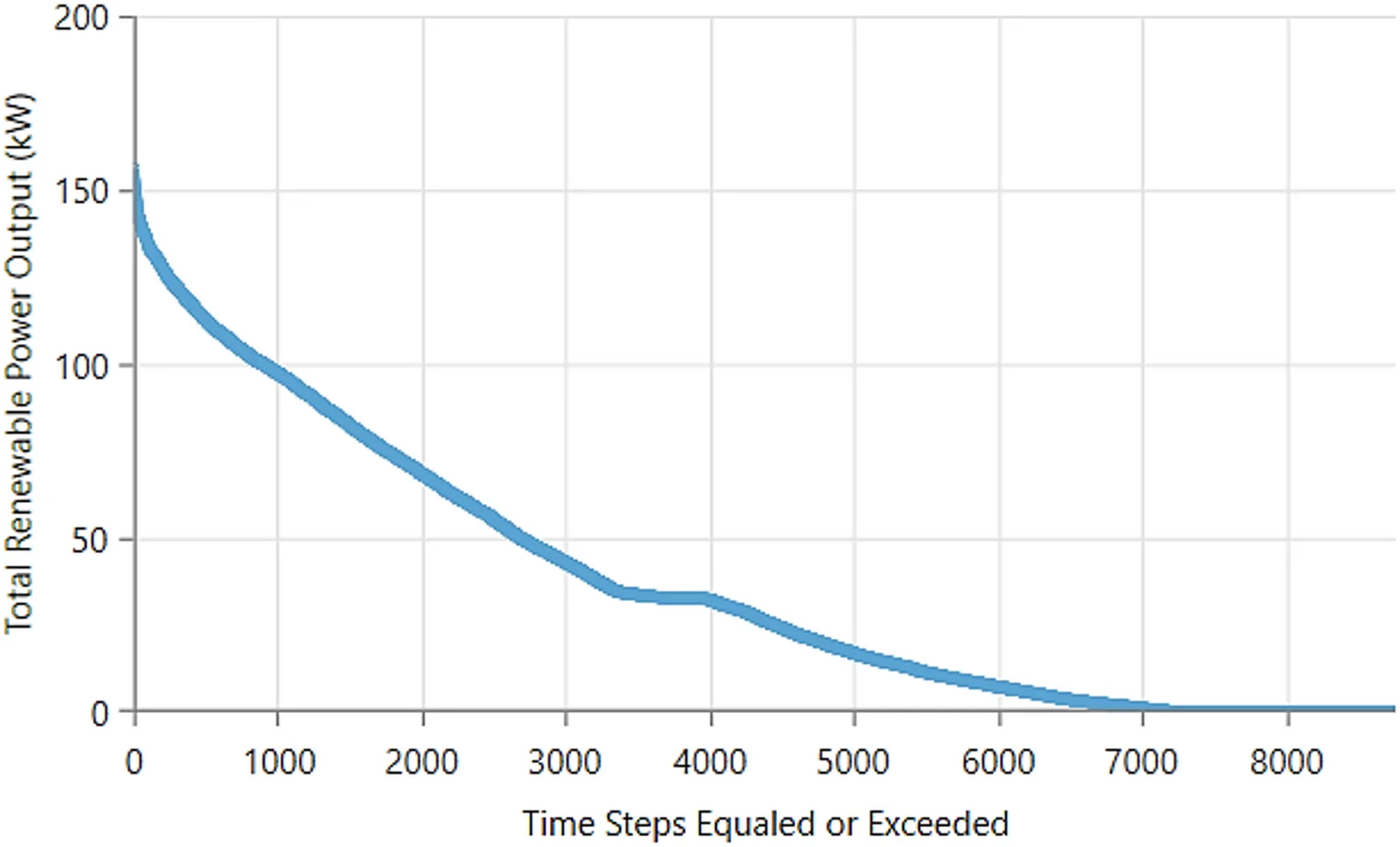
Duration curve representing the total renewable power output.

As indicated in Fig. 19 below, the curve shows the variation of renewable power output in descending order over a given period of time. From the curve above, it is clear that the power output of 155–160 kW was only available over a period of a very small number of hours. At any given time, the power output of renewable energy was over 90 kW only over 25% of the total period, whereas over 50% of the total period, the power output of renewable energy was over 45 kW. However, over a significant period of time during the year, the power output of renewable energy was relatively low, as indicated in the period of time during which the power output of renewable energy was below 20 kW over nearly 20% of the total period of operation, as indicated in the curve below. The curve indicates the intermittent nature of renewable energy, as indicated in the fact that over a small period of time, the power output of renewable energy was relatively high, whereas over a larger period of time, the power output of renewable energy was relatively low.

This confirms that the proposed hybrid configuration is the most suitable solution for reliable and sustainable market electrification under the given conditions.

### Emission reduction analysis

**Fig. 20:**
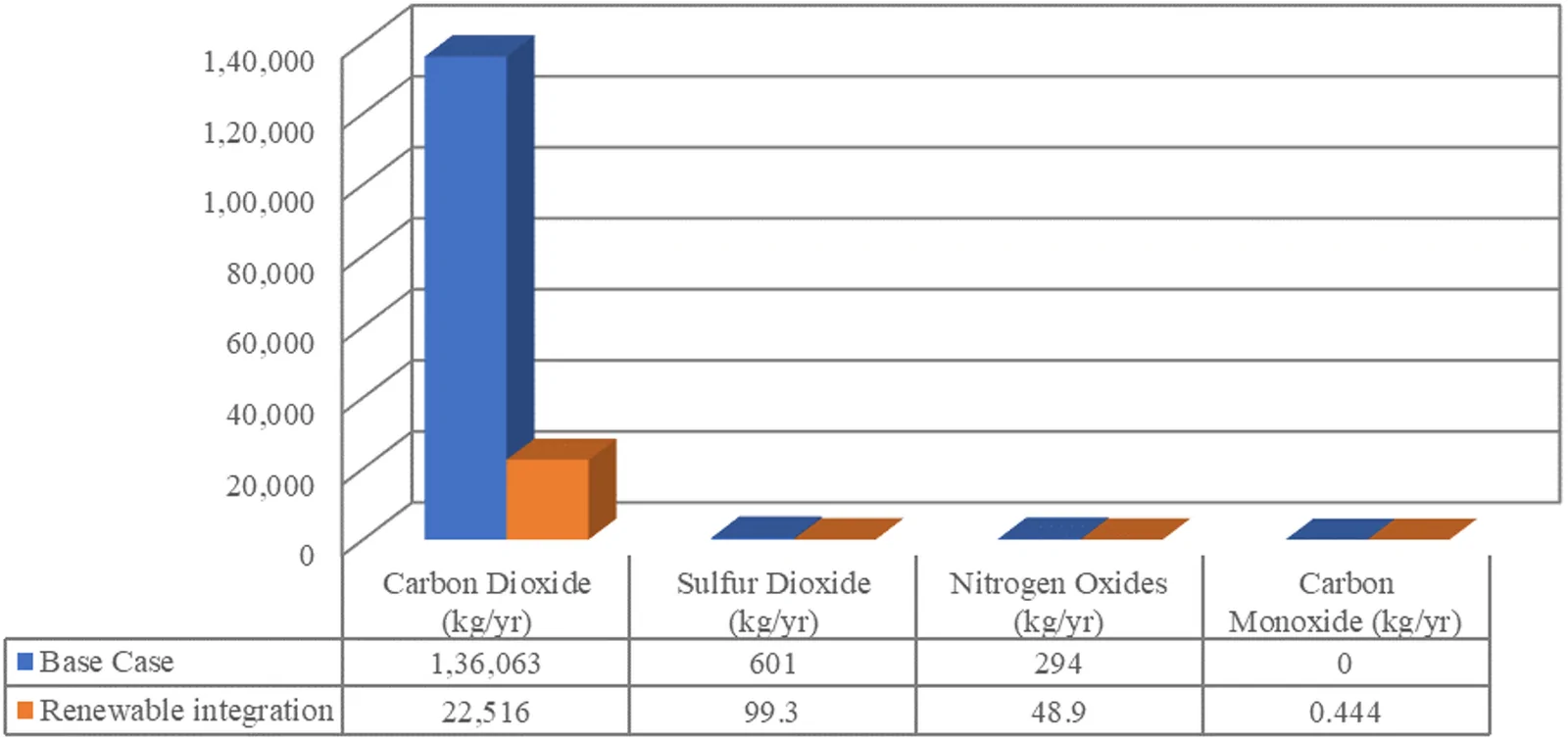
Comparison of emissions between the proposed microgrid system and the base case scenario.

Figure 20 presents the comparison of the yearly emissions of the designed microgrid and the base case microgrid. From the results presented above, it is clear that the incorporation of renewable energy sources into the microgrid system has the advantage of minimizing the emission of greenhouse gases and pollutants. For example, the amount of emitted carbon dioxide is minimized from 136,063 kg/yr in the base case microgrid to 22,516 kg/yr in the designed microgrid. This implies that the percentage of reduction of the emission of carbon dioxide is 83.5%. Similarly, the emission of sulfur dioxide is minimized from 601 kg/yr to 99.3 kg/yr, whereas the emission of nitrogen oxides is minimized from 294 kg/yr to 48.9 kg/yr. In addition, the amount of emitted carbon monoxide in the designed microgrid is very low, only 0.444 kg/yr, whereas in the base case microgrid, the amount is negligible.

### Sensitivity analysis for the proposed microgrid configuration

Sensitivity analysis is performed to evaluate the robustness of the proposed hybrid microgrid under uncertainties specific to the Nazipur Noor market, where electricity demand is influenced by commercial operating hours, seasonal trading activity, and grid reliability variations. Given the market’s dependence on daytime commercial loads and partial grid availability, assessing system sensitivity is essential to ensure stable performance under fluctuating resource and economic conditions. The results of the sensitivity analysis indicate that wind speed, hub height, and electricity sellback price are the most influential parameters affecting system performance.

Each input variable is varied over the range of ± 20% and ± 40% about the base case value. As shown in Table 5, the method varies input variables in proportion to changes in solar radiation (3.00–7.00 kWh/m2/day), ambient temperature (15.56–36.29 °C), and wind speed (2.49–5.81 m/s) to reflect realistic variability in renewable resource availability. Economic uncertainties are represented by variability in the inflation rate (5.4–12.6), nominal discount rate (9–21), and price of power (0.072–0.168) and sellback (0.036–0.084). Effects due to grid variability are represented by variability in hub height (9.6–22.4 m), failure frequency (300–700), mean repair time (0.6–1.4), and repair time variation (30–70).

**Table 5:** Input variables considered for the sensitivity analysis and their corresponding values.

Input Sensitive Variable	Values
Solar radiation (kWh/m^2^/day)	3.00, 4.00, **5.00**, 6.00, 7.00
Temperature (ºC)	15.56, 20.74, **25.92**, 31.11, 36.29
Wind speed (m/s)	2.49, 3.32, **4.15**, 4.98, 5.81
Inflation Rate (%)	5.4, 7.2, **9**, 10.8, 12.6
Nominal Discount Rate (%)	9, 12, **15**, 18, 21
Power Price (USD/kWh)	0.072, 0.096, **0.12**, 0.144, 0.168
Sellback Rate (USD/kWh)	0.036, 0.048, **0.06**, 0.072, 0.084
Hub height (m)	9.6, 12.8, **16**, 19.2, 22.4
Grid Failure Frequency (per Year)	300, 400, **500**, 600, 700
Grid Mean Repair Time (hour)	0.6, 0.8, **1**, 1.2, 1.4
Grid Variation Repair Time (%)	30, 40, **50**, 60, 70

Note: The bold values represent the middle or base values for sensitivity analysis.

**Fig. 21:**
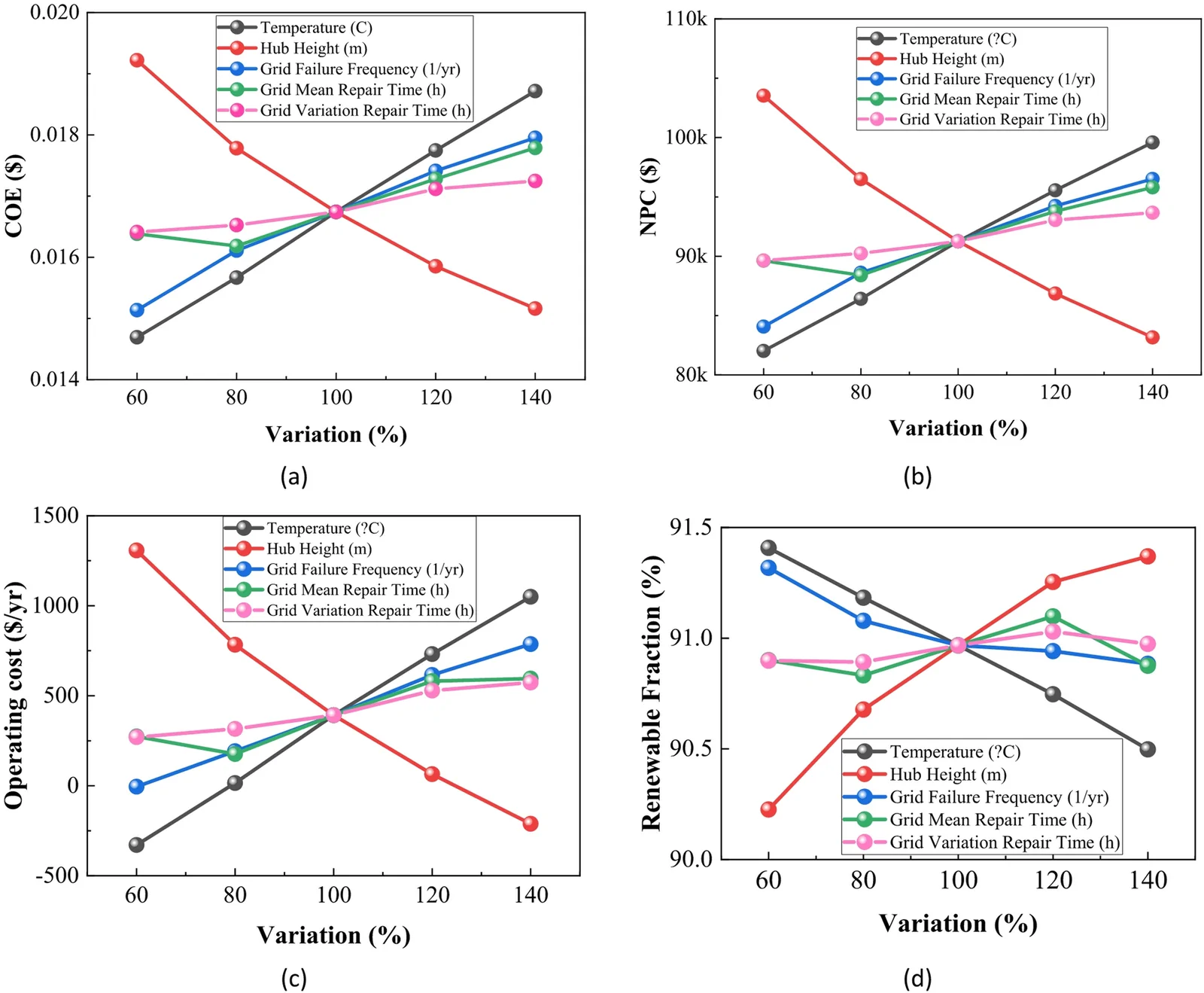
Effect of parameter variations on (**a**) COE, (**b**) NPC, (**c**) operating cost, and (**d**) RF.

Figure 21 Overall sensitivity analysis as shown in Fig. 13(a-d), the results demonstrate the sensitivity analysis of temperature, hub height, grid failure frequency, grid mean repair time, and grid repair time variation on the system’s COE, NPC, operating cost, and renewable fraction for the ± 40% variation range (60–140%). Figure 21(a) shows the COE increases with the rise in temperature from 0.01469 $/kWh at 60% temperature to 0.01871 $/kWh at 140%. On the other hand, increasing the hub height reduces the COE from 0.01922 $/kWh to 0.01516 $/kWh, showing enhanced wind energy harvesting capability. Grid-related parameters exhibit milder effects, with COE varying narrowly around 0.016–0.018 $/kWh. As shown in Fig. 21(b), the NPC follows a similar trend. NPC increases with temperature from 82,028 $ to 99,582.7 $, while higher hub height reduces NPC significantly from 103,529.5 $ to 83,169.4 $. Grid failure frequency and repair parameters cause moderate NPC variation, remaining within 84,075–96,508 $. Figure 21(c) indicates that operating cost is highly sensitive to temperature and hub height. Operating cost increases from − 328.96 $/yr to 1,049.66 $/yr with rising temperature, while higher hub height reduces operating cost sharply from 1,307.21 $/yr to − 211.30 $/yr, reflecting increased wind generation and grid export. Grid-related parameters again show limited influence. Finally, Fig. 21(d) shows that the renewable fraction decreases with temperature from 91.41% to 90.50%, while it increases with hub height from 90.23% to 91.37%. Variations due to grid reliability parameters remain minimal, staying close to the baseline value of 90.97%. Overall, the results confirm that temperature and hub height are the most influential sensitivity parameters, whereas grid repair-related variables have comparatively minor impacts due to effective renewable diversification and storage support.

The sensitivity ranking Table 6 shows a summary of the relative importance of infrastructure parameters on system performance. The results showed that hub height has the greatest sensitivity since increasing the height of the hub greatly reduces COE, NPC, and operation cost by harnessing wind energy. Grid mean repair time and temperature have a high sensitivity since prolonged outages coupled with higher temperatures increase economic costs while reducing renewable fraction. Grid failure frequency has a moderate sensitivity since it increases grid dependency while raising operation cost. Grid repair time variation has the lowest sensitivity since it causes a minor change in techno-economic and renewable performance parameters.

**Table 6:** Ranking of sensitive parameters based on their influence on output variations.

Rank	Parameter	Sensitivity Level	Metrics Affected	Reason
1	Hub Height	Very High	COE, NPC, Operating Cost, RF	COE decreases from 0.01922 to 0.01516 USD/kWh (≈ 21.1%), NPC decreases from 103,529.5 USD to 83,169.4 USD (≈ 19.7%), and operating cost decreases from 1,307.21 USD/yr to − 211.30 USD/yr.
2	Temperature	High	COE, NPC, Operating Cost, RF	COE increases from 0.01469 to 0.01871 USD/kWh (≈ 27.4%), NPC increases from 82,028.0 USD to 99,582.7 USD (≈ 21.4%), and renewable fraction decreases from 91.41% to 90.50%.
3	Grid Mean Repair Time	High	COE, NPC, Operating Cost	COE increases from 0.01638 to 0.01779 USD/kWh (≈ 8.6%), NPC increases from 89,628.6 USD to 95,805.2 USD (≈ 6.9%), and operating cost increases from 273.68 USD/yr to 595.14 USD/yr.
4	Grid Failure Frequency	Moderate	COE, NPC, Operating Cost	COE increases from 0.01514 to 0.01795 USD/kWh (≈ 18.6%) and NPC increases from 84,075.5 USD to 96,508.4 USD (≈ 14.8%).
5	Repair Time Variation	Low	COE, NPC, Operating Cost, RF	COE increases slightly from 0.01641 to 0.01725 USD/kWh (≈ 5.1%) and NPC increases from 89,643.3 USD to 93,672.9 USD (≈ 4.5%).

**Fig. 22:**
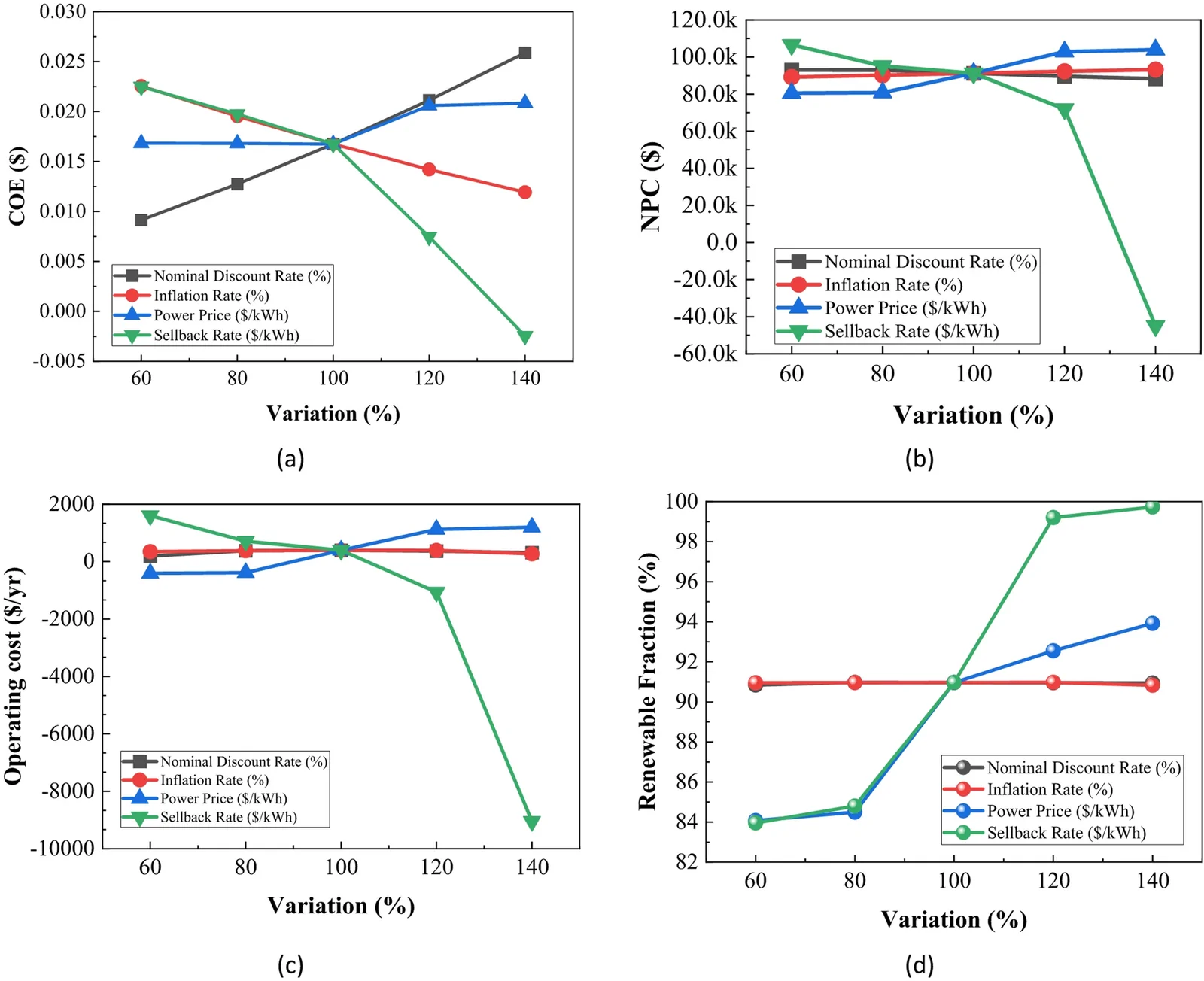
Influence of economic parameter variations on (**a**) COE, (**b**) NPC, (**c**) operating cost, and (**d**) RF.

The overall sensitivity analysis shown in Fig. 22 (a–d) presents the results of the impacts of ± 40% variation (60–140%) in nominal discount rates, inflation rates, power prices, and sellback rates on the performance. Figure 22 (a) presents the results where the COE increases as the nominal discount rate increases from 0.00915 to 0.02587 $/kWh. However, the inflation rate causes the COE to decrease from 0.02256 to 0.01193 $/kWh. The results show that the COE increases moderately as the power price increases from 0.01683 to 0.02084 $/kWh. However, the results show that the COE decreases sharply as the sellback price increases from 0.02250 to -0.00245 $/kWh. As shown in Fig. 22 (b), NPC decreases with higher nominal discount rate from 92,998.32 $ to 88,288.09 $, while it increases with inflation rate from 89,181.8 $ to 93,190.9 $ and power price from 80,519.15 $ to 103,925.1 $. In contrast, higher sellback rate causes NPC to decrease significantly from 106,578.9 $ to − 44,954.11 $. Figure 22 (c) indicates that operating cost increases with nominal discount rate from 192.86 $/yr to 310.97 $/yr and with power price from − 408.97 $/yr to 1,197.84 $/yr, while increasing sellback rate reduces operating cost sharply from 1,602.22 $/yr to − 9,050.03 $/yr. Finally, Fig. 22 (d) shows that renewable fraction increases with power price from 84.08% to 93.91% and with sellback rate from 83.96% to 99.73%, while remaining nearly constant for discount and inflation rate variations. Collectively, the results confirm that sellback rate and power price exert the strongest influence on economic and renewable performance, whereas discount and inflation rates have comparatively moderate effects.

As shown in the sensitivity ranking Table 7, the sellback rate has the highest influence on the economic parameters, significantly decreasing COE, NPC, and operating costs, while significantly increasing renewable fraction. Power price ranks the second most important parameter, as rising power prices increase system costs but also improve the fraction of renewables used. The nominal discount rate has a moderate influence, as it increases COE and operating costs, while decreasing NPC due to discounting effects. In contrast, the inflation rate shows the lowest sensitivity, as all performance metrics are only slightly affected. In total, revenue-related parameters are more important than macro-financial parameters in economic sensitivity assessment.

**Table 7:** Ranking of economic parameters based on their impact on output variation.

Rank	Parameter	Sensitivity Level	Metrics Affected	Reason
1	Sellback Rate	Very High	COE, NPC, Operating Cost, RF	COE decreases from 0.02250 to − 0.00245 $/kWh, NPC decreases from 106,578.9 to − 44,954.1 $, operating cost decreases from 1,602.22 to − 9,050.03 $/yr, and renewable fraction increases from 83.96% to 99.73%.
2	Power Price	High	COE, NPC, Operating Cost, RF	COE increases from 0.01683 to 0.02084 $/kWh, NPC increases from 80,519.2 to 103,925.1 $, operating cost increases from − 408.97 to 1,197.84 $/yr, and renewable fraction increases from 84.08% to 93.91%.
3	Nominal Discount Rate	Moderate	COE, NPC, Operating Cost	COE increases from 0.00915 to 0.02587 $/kWh, NPC decreases from 92,998.3 to 88,288.1 $, and operating cost increases from 192.86 to 310.97 $/yr.
4	Inflation Rate	Low	COE, NPC, Operating Cost	COE decreases from 0.02256 to 0.01193 $/kWh, NPC increases from 89,181.8 to 93,190.9 $, and operating cost varies narrowly around 345–380 $/yr.

**Fig. 23:**
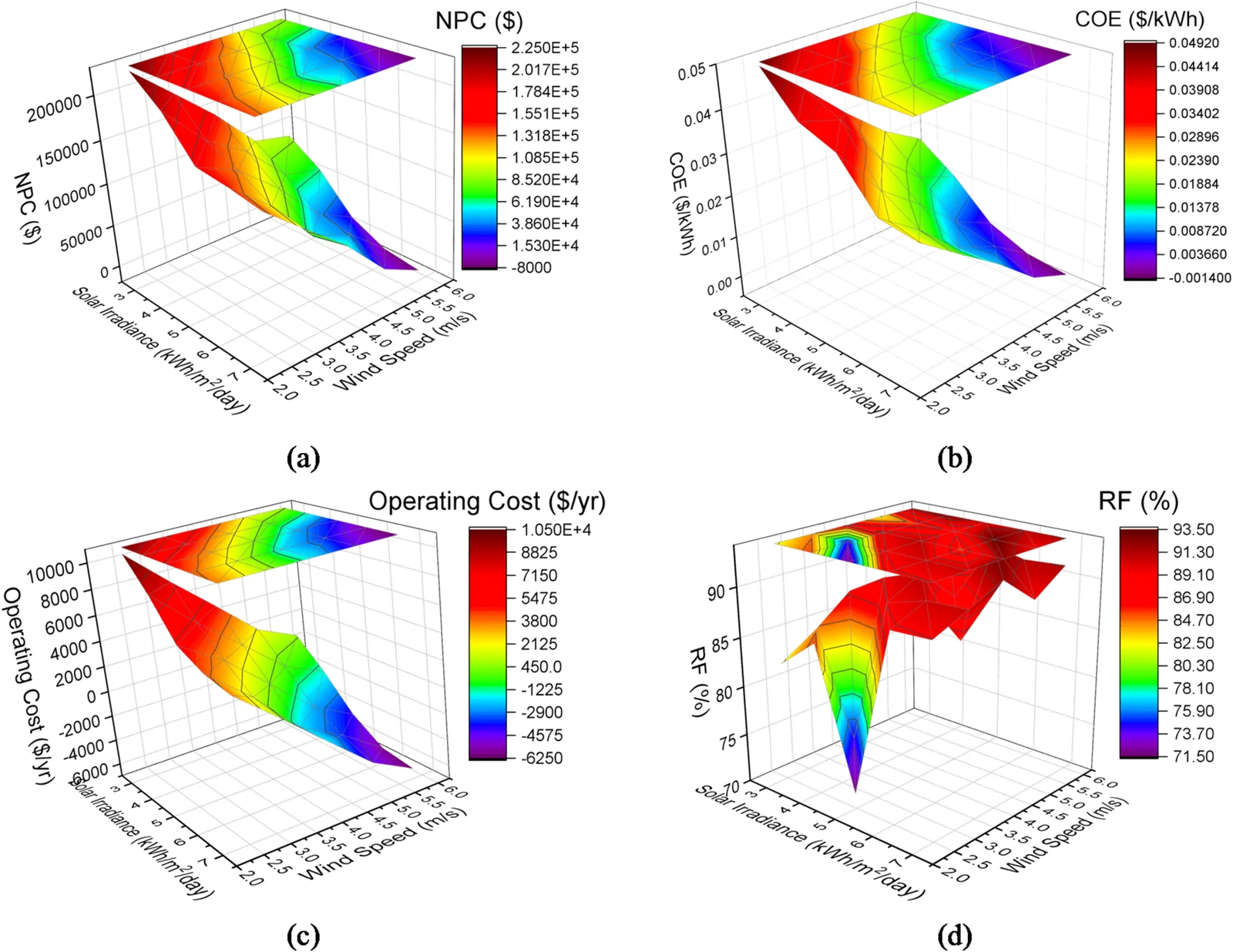
Response surfaces of techno-economic and sustainability metrics to solar irradiance and wind speed.

Figure 23 presents the response surfaces of key techno-economic and sustainability indicators as functions of solar irradiation (2.998–6.995 kWh/m²/day) and wind speed (2.4915–5.8135 m/s). Figure 23 (a) shows that NPC decreases consistently with increasing wind speed, regardless of solar level. At low solar and wind conditions (2.998 kWh/m²/day, 2.4915 m/s), NPC is highest at USD 224,440.8, while at high solar and wind levels (6.9953 kWh/m²/day, 5.8135 m/s), NPC decreases to − USD 7,743.6, indicating strong revenue from surplus generation. Increasing solar irradiation further reduces NPC, particularly at higher wind speeds. Figure 23 (b) illustrates that COE decreases sharply with increasing wind speed and solar irradiation. COE drops from 0.04912 USD/kWh at low solar–wind conditions to − 0.00134 USD/kWh at the highest solar and wind combination, confirming the strong cost-reduction effect of resource availability. As shown in Fig. 23 (c), operating cost declines significantly with increasing wind speed. Operating cost is highest at USD 10,488.36/yr under low solar–wind conditions and becomes strongly negative (− USD 6,227.02/yr) at high solar and wind levels, reflecting net revenue from electricity export. Figure 23 (d) presents the renewable fraction, which generally increases with both solar irradiation and wind speed. Renewable fraction rises from 82.17% at low resource levels to values above 91% at higher wind speeds, reaching a maximum of 91.40% at 6.9953 kWh/m²/day and 5.8135 m/s. Overall, the response surfaces confirm that wind speed exerts the strongest influence, while increased solar irradiation further enhances economic and sustainability performance.

As shown in the sensitivity ranking Table 8, indicates that wind speed is the most influential renewable parameter, as increasing wind speed consistently decreases COE, NPC, and operating cost while significantly increasing renewable fraction, even leading to negative costs under high-resource conditions. Solar irradiation ranks second, as higher solar levels also improve all performance metrics, though with a comparatively smaller impact than wind speed. Overall, the response-surface analysis confirms that wind resource availability dominates system economics and sustainability, while solar irradiation plays a complementary but secondary role in enhancing system performance.

**Table 8:** Sensitivity ranking of renewable resource parameters based on response surface analysis.

Rank	Parameter	Sensitivity Level	Metrics Affected	Reason
1	Wind Speed	Very High	COE, NPC, Operating Cost, RF	COE decreases from 0.04912 to −0.00134 $/kWh, NPC decreases from 224,440.8 USD to −7,743.6 USD, operating cost decreases from 10,488.36 USD/yr to −6,227.02 USD/yr, and renewable fraction increases from ~82% to >91%.
2	Solar Irradiation	High	COE, NPC, Operating Cost, RF	COE decreases from 0.04912 to 0.02441 $/kWh at low wind and further at higher wind speeds; NPC decreases from 224,440.8 USD to 137,455.3 USD at low wind; operating cost decreases from 10,488.36 USD/yr to 3,653.85 USD/yr; renewable fraction increases from 82.17% to ~90.54%.

### Correlation analysis of microgrid variables

**Fig. 24:**
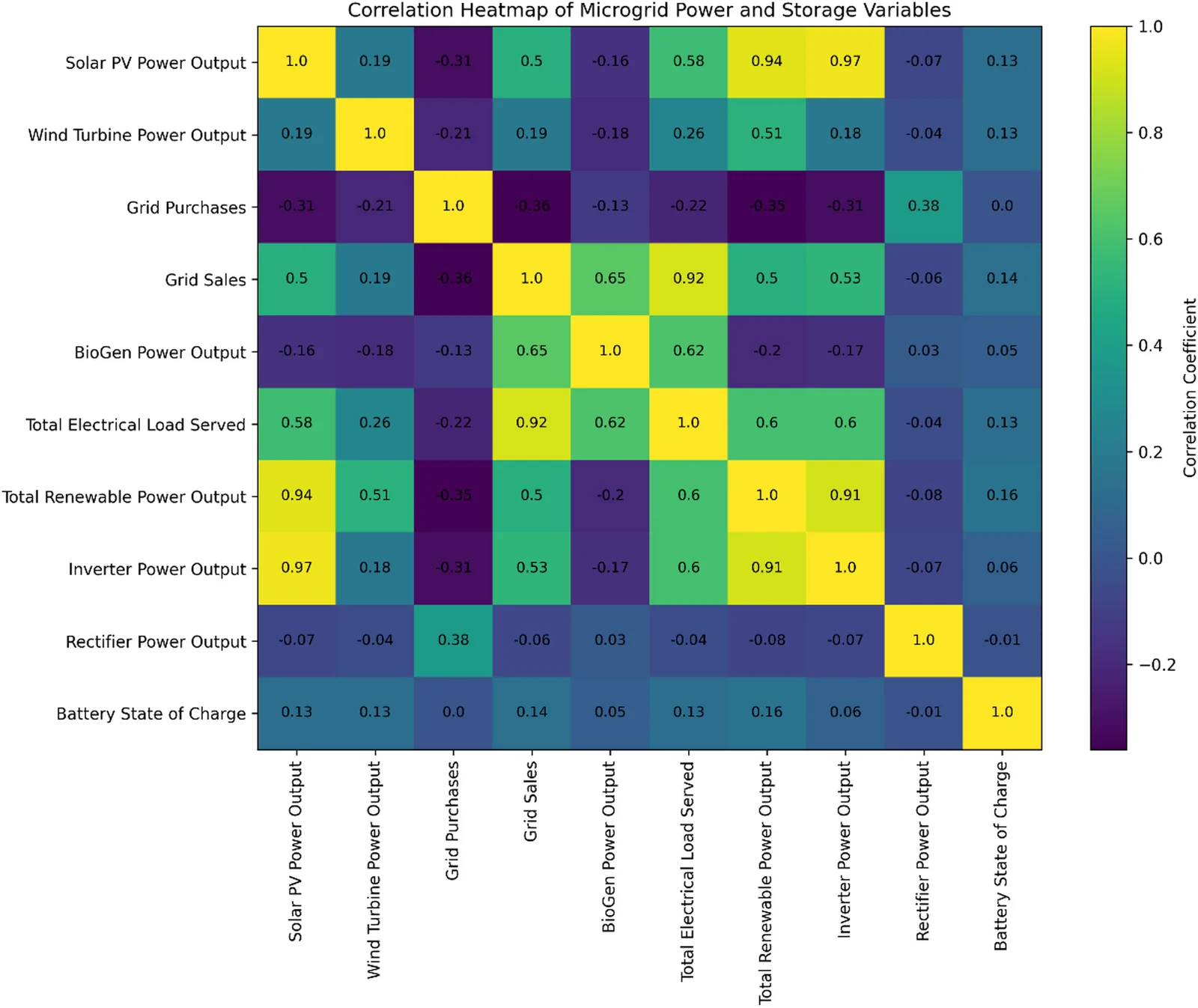
Correlation heatmap of microgrid operational variables.

As presented in Fig. 24, the pairwise linear relationship between key power flow variables of the proposed hybrid microgrid is presented in terms of correlation coefficients from − 1 to + 1. High positive correlations are found between solar PV power output and inverter power output (0.97) and between solar PV power output and total renewable power output (0.94), indicating that solar PV generation is dominant in renewable generation and inverter operation. Grid sales are highly correlated with total electrical load served (0.92) and moderately correlated with biogas power output (0.65), indicating increased grid sales in periods of high generation. Total renewable power output is highly correlated with inverter power output (0.91) and moderately correlated with total load served (0.60). On the other hand, grid purchases are negatively correlated with renewable outputs such as -0.35 with total renewable power output, indicating decreased grid purchases in periods of high renewable generation. Battery state of charge is found to be weakly correlated with all variables (< 0.16), indicating that battery operation is relatively stable with no significant dependence on instantaneous power flow variables.

The weak correlation of the battery state of charge with other system variables can be attributed to its limited operational role in the optimized configuration. As observed from the SOC profile, the battery operates within a relatively narrow range (approximately 88%–100%), indicating limited charging–discharging activity. This behavior suggests that the optimized system primarily relies on renewable generation and grid interaction, with the battery serving mainly as a backup and balancing component to ensure supply reliability during short-term fluctuations and renewable intermittency. Although a smaller battery capacity could potentially increase utilization through deeper charge–discharge cycles, the selected battery size was determined through HOMER Pro techno-economic optimization to maintain operational reliability and system resilience under varying load and resource conditions.

The specific purpose and objective of the research are related to the designing and evaluation of a grid-connected hybrid renewable microgrid system based on the characteristics of the demand profile of the specific local markets in the rural areas of Bangladesh, where the demand profile of the electricity sector is largely influenced by the characteristics of the commercial activities and exhibits a very dissimilar profile in comparison with the conventional demand profile of the residential sector. Hence, this research work is useful in providing a viable solution to achieve sustainable electrification of markets.

Despite its advantages, the use of HOMER Pro involves certain limitations. The software relies on predefined component models and simplified control strategies, which may not fully capture dynamic system behavior and real-time operational constraints. In addition, advanced optimization techniques such as multi-objective evolutionary algorithms or real-time control strategies are not directly implemented within the platform. Furthermore, the accuracy of results depends on the quality and resolution of input data, particularly load profiles and resource availability. Therefore, future work may incorporate hybrid approaches combining HOMER Pro with advanced optimization algorithms or real-time simulation tools to enhance modeling accuracy and system performance evaluation.

## Conclusion

In the present research work, a detailed techno-economic analysis and a detailed analysis of the environmental impacts of a hybrid renewable microgrid for local market electrification in a rural region of Bangladesh with a case study of the Nazipur Noor market have been conducted. A realistic load model of a local market with a highly concentrated load during the daytime along with coincidence and seasonal variation, which is normally overlooked in microgrid research work related to residential areas, has been considered in the present research work. After conducting a detailed analysis of a hybrid renewable microgrid with HOMER Pro software with a total of 1,056 different configurations, a hybrid microgrid with a configuration of a PV-WT-BioGen-BESS-Grid (Case I) was found to be the optimal solution with a lower NPC of USD 91,255.5 along with a lower COE of USD 0.0167/kWh and a higher percentage of renewable energy penetration at 90.97%. The major share of electricity generated annually is contributed by solar PV systems with 48.4% or 209,060 kWh, followed by wind energy systems with 25.9% or 111,733 kWh, and then biogas systems with 17.2% or 74,122 kWh, while grid purchase contributes only 8.51% to the total demand. From the environmental assessment table, there are significant environmental benefits to be achieved, such as a reduction in CO2 emissions by 83.5% from 136,063 to 22,516 kg/yr in the base case and in the proposed case, respectively. Sensitivity analysis and response surface showed that wind speed, hub height, and electricity sellback rate are major parameters that greatly impact the economic viability of wind and solar systems, while grid parameters are less significant due to effective diversification of renewable resources.

The future direction for further research would be to extend the optimization framework beyond the HOMER-based enumeration and apply other advanced optimization techniques such as genetic algorithms, particle swarm optimization, differential evolution, and multi-objective evolutionary optimization techniques. In addition, the high-fidelity modeling and simulation would be carried out in the MATLAB/Simulink environment for the analysis of dynamic system behavior, voltage stability, and frequency stability. At the same time, other techniques such as stochastic modeling, machine learning prediction, and uncertainty quantification based on the Python programming environment would be applied for the optimization methods.

## Data Availability

The data that is used for the study is made available by the corresponding author of the study upon reasonable request.
